# Four polyopisthocotyleans (Platyhelminthes: Monogenea) from carangid fishes in the Mediterranean, off the Algerian coasts

**DOI:** 10.1016/j.crpvbd.2021.100026

**Published:** 2021-05-04

**Authors:** Chahinez Bouguerche, Fadila Tazerouti, Jean-Lou Justine

**Affiliations:** a Université des Sciences et de la Technologie Houari Boumediene, Faculté des Sciences Biologiques, Laboratoire de Biodiversité et Environnement: Interactions - Génomes, BP 32, El Alia Bab Ezzouar, Alger, Algeria; b Institut Systématique Évolution Biodiversité (ISYEB), Muséum National d’Histoire Naturelle, CNRS, Sorbonne Université, EPHE, Université des Antilles, 57 rue Cuvier, CP 51, 75005 Paris, France

**Keywords:** Monogenea, Polyopisthocotylea, Mediterranean, Taxonomy, Teleosts

## Abstract

Four polyopisthocotyleans were collected from the gill filaments of carangids from off the Algerian coast, southern Mediterranean. Specimens of *Gastrocotyle trachuri* van Beneden & Hesse, 1863 (Gastrocotylidae) and *Cemocotyle* cf. *trachuri* Dillon & Hargis, 1965 (Heteraxinidae) from the Mediterranean horse mackerel *Trachurus mediterraneus* (Steindachner), *Zeuxapta seriolae* (Meserve, 1938) (Heteraxinidae) from the greater amberjack *Seriola dumerili* (Risso) and *Pyragraphorus hollisae* Euzet & Ktari, 1970 (Pyragraphoridae) from the pompano *Trachinotus ovatus* (Linnaeus) are redescribed based on newly collected specimens. Their taxonomically important morphological features (male copulatory organ and clamp sclerites) are described and illustrated, and the morphometric variation between Mediterranean and oceanic specimens is highlighted. Careful examination of the specimens of *Cemocotyle* Sproston, 1946 from the Mediterranean revealed that they exhibited unusual features compared with *Cemocotyle trachuri* Dillon & Hargis, 1965 from the Pacific, mainly the absence of the terminal lappet, thus questioning previous records of this species in the Mediterranean. New geographical locality records are provided for *Z. seriolae* and *P. hollisae*. The presence of *C.* cf. *trachuri* and *Z. seriolae* in the Mediterranean is noteworthy as these monogeneans were initially described in the Pacific Ocean. This study extends the geographical range of *Z. seriolae* to the southern Mediterranean.

## Highlights

• *Gastrocotyle trachuri*, *Cemocotyle* cf. *trachuri*, *Zeuxapta seriolae* and *Pyragraphorus hollisae* are redescribed.
• Summarized host and distribution data of the species and comments on their taxonomy and host specificity are provided.
• Algeria is a new geographical record for *Z. seriolae* and *P. hollisae.*
• *Z. seriolae* is reported for the first time in the South-Western Mediterranean.
• Unusual features of the new specimens of *Cemocotyle* question previous records of *Cemocotyle trachuri* in the Mediterranean.

## Graphical abstract

**Figure:**
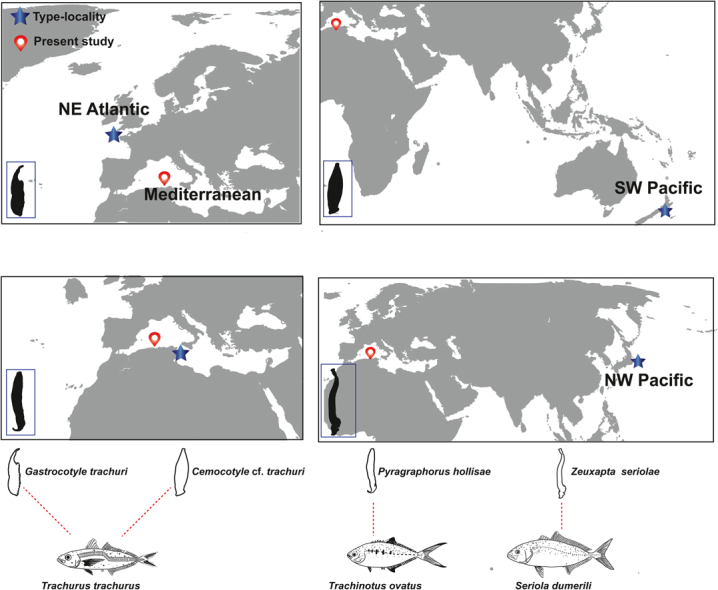

## 1 Introduction

Monogeneans of Mediterranean fishes were among the first to have been depicted and described (Ulmer & James, 1981). Early studies on monogeneans from the North-Western Mediterranean Sea include those by Professor Louis Euzet and colleagues, who made significant contributions providing a solid ground for later studies (Euzet, 1957; Euzet & Audouin, 1959; Euzet & Razarihelisoa, 1959; Euzet & Trilles, 1961; Euzet & Ktari, 1971; Euzet & Suriano, 1973). Research efforts on monogeneans flourished as well in the South-Western Mediterranean and in the French-speaking Africa, throughout training of local researchers by Professor Euzet and parasitologists of his ‘school’ (Scholz et al., 2018), mainly Claude Combes (see e.g. Kechemir, 1978) and Jean-Lou Justine (see Chaabane et al., 2015, 2016a, b; Kheddam et al., 2016; Ayadi et al., 2017; Chaabane et al., 2017; Bouguerche et al., 2019a, b, c; Bouguerche et al., 2020a, b; Kheddam et al., 2020; Azizi et al., 2021).

In Algeria, as of the year 1995, early efforts of Dr Faiza Amine contributed significantly to the systematics of some monogeneans from off the Algerian coast (Kouider El Ouahed-Amine, 1998; Amine & Euzet, 2005; Amine et al., 2006a, b, 2007a, b). Nonetheless, regardless of the growing number of possible hosts, surveys of monogenean parasites of marine fishes from off the Algerian coast remain relatively sparse and targeted mostly and largely ecology (Kaouachi et al., 2010; Marzoug, 2012; Ramdane et al., 2013; Brahim Tazi et al., 2016; Benhamou et al., 2017; Hadjou et al., 2017; Ichalal et al., 2017; Ider et al., 2018). Moreover, these surveys focused on economically important host species, mostly sparids (Kouider El Ouahed-Amine, 1998; Kaouachi et al., 2010; Marzoug, 2012; Ramdane et al., 2013; Benhamou et al., 2017; Ider et al., 2018). In addition, most of these data were published in local scientific journals or remain unpublished in MSc and PhD theses, making them difficult to access. It is also likely that some of the monogeneans included within these studies have been misidentified.

Amongst teleost fishes frequently encountered in Algerian marine waters, carangid fishes represent suitable candidates for taxonomic surveys of parasites. They include 146 species of 32 genera (Froese & Pauly, 2016), are largely distributed worldwide, and occur in both tropical and temperate waters (Fischer et al., 1987; Smith-Vaniz, 1999). Estimation of parasite biodiversity supported by these fishes is far from being completed, as most of the implicated fish hosts are with a cosmopolitan distribution and a migratory life-style. Hence, both morphological and molecular data on their parasite fauna, along with type- and voucher specimens in recognised museum collections must be provided for each locality to assure unbiased descriptions of new species.

During a survey of helminth parasites of marine fishes from the South-Western (SW) Mediterranean, six monogenean species were found on the gills of carangids. The Mediterranean horse mackerel *Trachurus mediterraneus* (Steindachner) distributed in the North-East (NE) Atlantic and the Mediterranean was found parasitized by four species: *Pseudaxine trachuri* Parona & Perugia, 1890, *Allogastrocotyle trachuri* Nasir & Fuentes Zambrano, 1983, *Cemocotyle* cf. *trachuri* Dillon & Hargis, 1965 and *Gastrocotyle trachuri* van Beneden & Hesse, 1863; the pompano *Trachinotus ovatus* (L.) which occurs in the NE Atlantic from Africa to the North Sea, was found parasitized by the rare species *Pyragraphorus hollisae* Euzet & Ktari, 1970; and the cosmopolitan species *Seriola dumerili* (Risso) which is found in the Pacific, Atlantic and Indian oceans, and the Mediterranean, harboured a single species, *Zeuxapta seriolae* (Meserve, 1938). The first two monogenean species were redescribed recently (Bouguerche et al., 2019b, c). Herein, we provide detailed illustrated descriptions of the remaining four polyopisthocotylean monogeneans and discuss their taxonomic status, hosts, and distribution.

## 2 Materials and methods

Freshly-caught fishes collected off the Algerian coast at Cherchell (36°36′31″N, 2°11′50″E) and Bouharoun (36°37′24″N, 2°39′17″E) were purchased from local fishermen. All carangids were relatively young specimens, far from the maximum lengths reported for these species (Smith-Vaniz, 1999). Fishes were transferred to the laboratory shortly after capture and identified using keys (Fischer et al., 1987; Smith-Vaniz, 1999). Gills were removed from each fish and observed under a stereomicroscope (Carl Zeiss™ Stemi™ DV4 Stereomicroscope, Germany) for the presence of monogeneans. The following fish species were examined: *Trachurus mediterraneus* (total length (TL) 20–26 cm, *n* = 256), *Trachinotus ovatus* (TL 25–45 cm, *n* = 36), and *Seriola dumerili* (TL 40–42 cm, *n* = 2).

Monogeneans were removed alive from gills using fine dissection needles, then preserved in 70% ethanol, stained with acetic carmine, dehydrated in ethanol series (70, 96 and 100%), cleared in clove oil, and mounted in Canada balsam. Some specimens were mounted in Berleseʼs fluid to study the morphology of the clamps and the genital atrium. Drawings were made with the aid of a Leitz microscope equipped with a drawing tube (Leitz, Wetzlar, Germany). Drawings were scanned and redrawn on a computer with Adobe Illustrator (CS5). Measurements are in micrometres and are indicated as the range followed by the mean and the number of measurements in parentheses.

## 3 Results

### *3.1* *Gastrocotyle trachuri* van Beneden & Hesse, 1863

#### 3.1.1 Taxonomic summary

*Type-host*: *Trachurus trachurus* (L.) (syn. *Caranx trachurus* (L.), WoRMS, 2021), Atlantic horse mackerel [1–12, 15–22, 24, 25, 27, 29–31, 32, 34, 36–38, 40, 41, 43, 44, 47, 48; present study].

*Other hosts*: *Trachurus mediterraneus* (Steindachner) [28, 30, 33, 45, 49]; *T. picturatus* (Bowdich) [19, 24, 36, 38, 43, 49]; *T. lathami* Nichols [23, 42]; *T. novaezelandiae* Richardson [13]; *T. indicus* (Necrasov) [15]; *T. capensis* Castelnau [19, 24, 46]; *T. trecae* Cadenat [19]; *Trachurus* spp. [35]; *Selar crumenophthalmus* Bloch [14, 15]; *Decapterus* sp. [15]; *Decapterus russelli* (Rüppell) [26]; *D. maruadsi* (Temminck & Schlegel) [26]; *Selar boops* (Cuvier) [26].

*Type-locality*: North-East (NE) Atlantic, off France (Brest) [1].

*Additional localities*: Atlantic [1, 3–5, 8, 10–12, 18–25, 27, 31, 32, 36–38, 41–44, 46, 49]; Mediterranean [2, 9, 16, 17, 28, 30, 33, 34, 36–38, 40, 45, 47–49; present study]; Pacific [6, 7, 13–15, 35]; Indian Ocean [15, 26, 29].

*References*: [1] van Beneden & Hesse (1863); [2] Parona & Perugia (1889); [3] Nicoll (1914); [4] Baylis & Jones (1933); [5] Jones (1933); [6] Yamaguti (1938); [7] Yamaguti (1942); [8] Sproston (1946); [9] Palombi (1949); [10] Llewellyn (1956); [11] Llewellyn (1959); [12] Llewellyn (1962); [13] Lebedev (1968); [14] Yamaguti (1968); [15] Parukhin (1976); [16] Lambert (1978); [17] Orecchia & Paggi (1978); [18] Gaevskaya & Kovaleva (1979); [19] Shaw (1979); [20] Gaevskaya & Kovaleva (1980); [21] Piasecki (1982); [22] Llewellyn (1983); [23] Nasir & Fuentes Zambrano (1983); [24] Gaevskaya & Kovaleva (1985); [25] Rego (1987); [26] Parukhin (1988); [27] López-Román & De Armas Hernández (1989); [28] Radujkovic & Euzet (1989); [29] Reimer (1990); [30] Euzet et al. (1993); [31] Naidenova & Mordvinova (1997); [32] Palm et al., (1999); [33] Mollaret et al. (2000); [34] Jovelin & Justine (2001); [35] Zhang et al. (2003); [36] MacKenzie et al. (2004); [37] Campbell (2008); [38] MacKenzie et al. (2008); [39] Fernandez-Jover et al. (2010); [40] Strona et al. (2010); [41] Ângelo (2011); [42] Braicovich et al. (2012); [43] Costa et al. (2012); [44] Rahemo (2012); [45] Akmirza (2013); [46] Le Roux (2013); [47] Feki et al. (2016); [48] Ichalal et al. (2017); [49] Hamdi et al. (2019).

*Descriptions*: [1, 2, 5–9, 13, 14, 21, 23, 28, 41, 46; present study].

*Site on host*: Gills.

*Voucher material*: 38 voucher specimens are deposited in the collections of the Muséum National d’Histoire Naturelle, Paris, France (MNHN HEL1500–HEL1544).

#### 3.1.2 Description

**Fig. 1:**
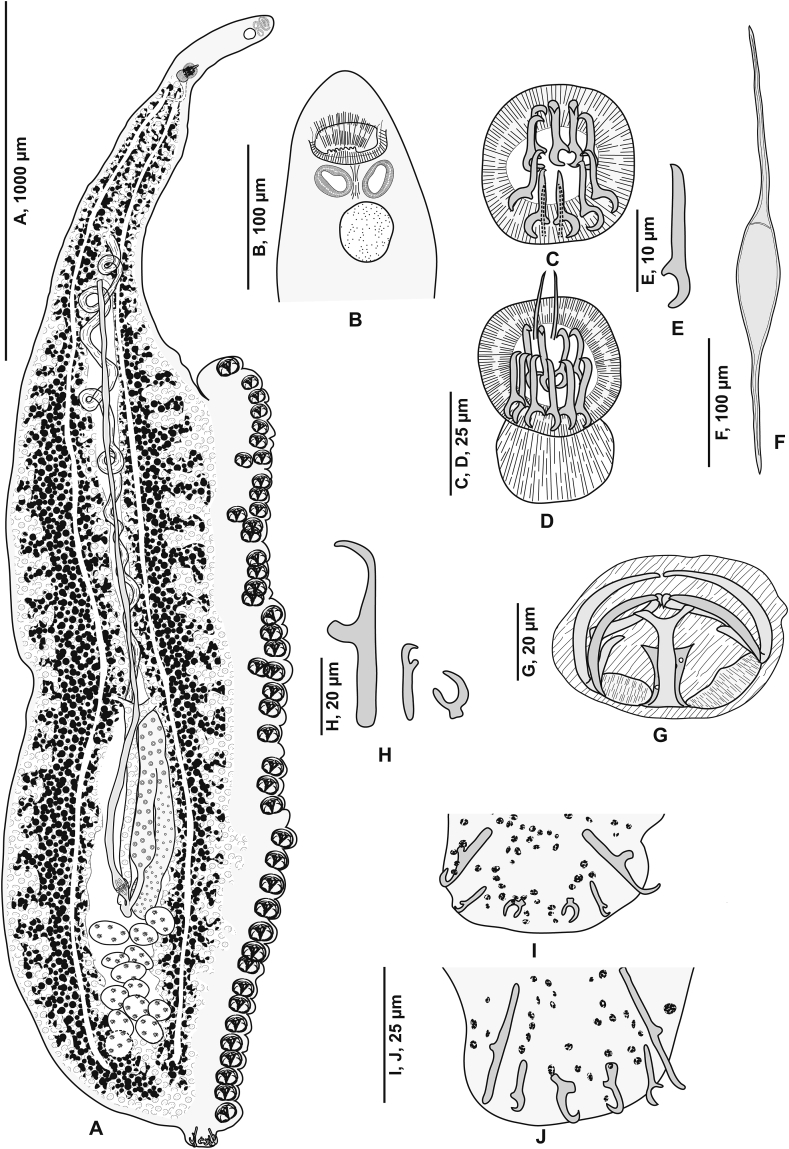
*Gastrocotyle trachuri* ex *Trachurus mediterraneus*. **A** Body, total view (MNHN HEL1505). **B** Anterior extremity showing the relative position of prohaptoral suckers, pharynx and male copulatory organ (MNHN HEL1506). **C** Male copulatory organ, penis invaginated (MNHN HEL1504). **D** Male copulatory organ, penis evaginated (MNHN HEL1506). **E** Atrial hook (MNHN HEL1506). **F** Egg (MNHN HEL1501). **G** Clamp, ventral view (MNHN HEL1502). **H** Uncini of terminal lappet (MNHN HEL1503). **I**, **J** Posterior lappet (MNHN HEL1503).

**Fig. 2:**
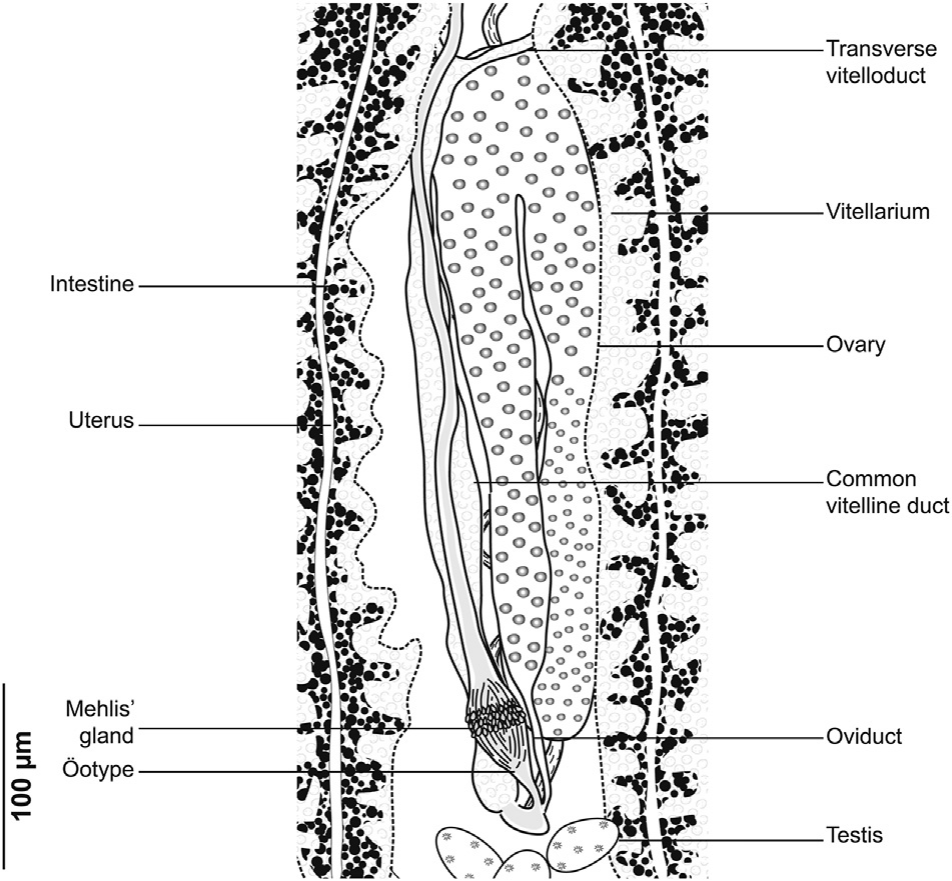
*Gastrocotyle trachuri* ex *Trachurus mediterraneus*. Detail of the reproductive organs in the region of ovary (MNHN HEL1501).

**Fig. 3:**
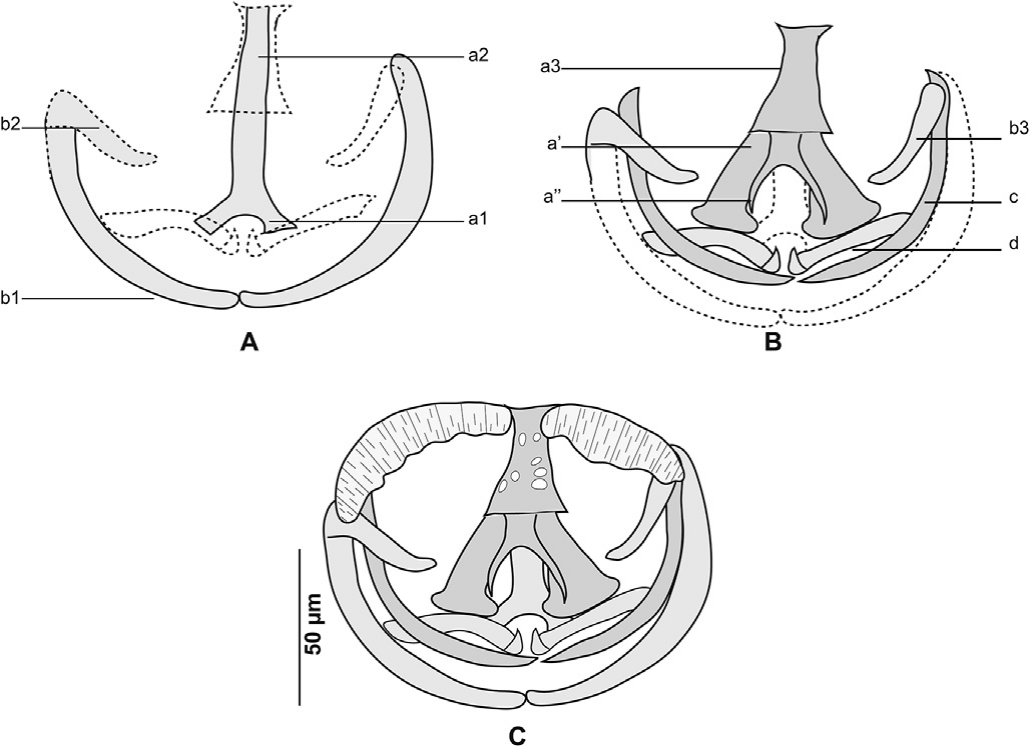
*Gastrocotyle trachuri* ex *Trachurus mediterraneus*. **A** Dorsal jaw. **B** Ventral jaw. **C** Clamp, dorsal view (MNHN HEL1500).

[Based on 26 specimens; Fig. 1, Fig. 2, Fig. 3; Table 1] Body elongated, narrow, symmetrical in first anterior third and considerably wider and asymmetrical posteriorly. Haptor parallel to longitudinal body axis, occupying 2/3 of total body length. Haptor with single row of clamps (Fig. 1A) and terminal lappet (Fig. 1I, J). Terminal lappet armed with 6 uncini; lateral pairs large and stout, medial pairs small (Fig. 1H). Clamps of *Gastrocotyle* type (Fig. 1G). Ventral arm of median spring *a1* Y-shaped, long, distal part of *a1* Y-shaped, with short branches of equal size, each limb abutting on short oblique sclerites. Dorsal arm of median spring *a3* shorter than *a1*, distally broad, with 3–4 pairs of apertures arranged in 2 longitudinal symmetrical parallel rows, distal end of *a1* with 2 superposed accessory skeletal pieces at its distal end: *a’* represented by V-shaped process; *a”* (Fig. 3A). Ventral arm of ventral jaw sclerites *b1,* dorsal arm *b2* short and curved inwards, *b2* not reaching accessory skeletal piece of dorsal arm of median spring (Fig. 3B). Dorsal jaw sclerites *c* shorter than ventral, *c* reaching midline on distal side. Oblique sclerites *d* long, with inner ends folded inwards. Muscle connecting *a2* and *b2* present on proximal side (Fig. 3C).

**Table 1:** Measurements of *Gastrocotyle trachuri* van Beneden & Hesse, 1863 from various localities

Host	*Trachurus trachurus*	*T. capensis*	*T. mediterraneus*	*T. novaezelandiae*	*Decapterus* sp.	*T. trachurus*
Locality	SW Mediterranean, off Algeria	SE Atlantic, off Namibia	Central Mediterranean, off Montenegro	SE Pacific, off Australia, the Tasman Sea	Western Indian Ocean, off India	NE Atlantic, off Plymouth
Source	Present study	Piasecki (1982)	Radujkovic & Euzet (1989)	Lebedev (1968)	Pillai (1968)	Jones (1933)
Body length	2,370–3,675 (3,040; *n* = 13)		2,000–3,000	2,690–5,030	2,200–3,100	4,700
Haptor length	1,620–2,770 (2,140; *n* = 13)					
Body width	610–1025 (775; *n* = 14)		1,000	560–730	500–750	1,200
Clamp number	30–40 (35; *n* = 16)	33	25–40	20–24	22–30	32–40
Clamp length	60–75 (65; *n* = 16)	70–100	50–70^a^	60–80	45–50	80^a^
Clamp width	38–58 (45; *n* = 14)	55–77		50–60	60–75	
Postero-lateral hooks length	16–20 (18; *n* = 15)	25	10	17–19	18–20	
Hamulus length	35–52 (46; *n* = 9)	58	50	42–53	42–45	
Posterior hooks length	20–26 (23; *n* = 22)		20	21–24	22–25	
Prohaptoral sucker length	22–33 (27; *n* = 10)					23
Prohaptoral sucker width	26–39 (31; *n* = 8)					15
Pharynx length	34–54 (43; *n* = 8)					46
Pharynx width	30–50 (38; *n* = 7)					30
Distance genital atrium to anterior extremity	185–385 (250; *n* = 22)				120–250	
Genital atrium length	25–30 (28; *n* = 22)	16^a^		14–43	30–35	23^a^
Genital atrium width	22–33 (27; *n* = 22)			10–30	35–35	
Number of atrial hooks	12 (*n* = 26)	12	12	16	12	12
Atrial hooks length	18–19 (18; *n* = 26)		20	14	12–20	
Number of testes				10–11		
Egg length	270–315 (283; *n* = 10)	250				
Egg width	65–90 (78; *n* = 10)					

*Abbreviations*: NE, North-Eastern; SE, South-Eastern; SW, South-Western.

a Diameter.

Prohaptoral suckers 2, oval, muscular, opening laterally (Fig. 1B), aseptate. Pharynx voluminous, spherical. Intestinal bifurcation posterior to genital atrium. Caeca with numerous lateral and axial diverticula.

Testes *c*.13 in number, small, follicular, in intercaecal field of posterior body third, often obscured by vitellarium. Vas deferens conspicuous, dorsal to uterus, running forward along body midline, expanding in its terminal part into ejaculating bulb (Fig. 1D). Male copulatory organ composed of genital atrium and stylet (Fig. 1C). Genital atrium muscular, mid-ventral, opening at short distance from anterior extremity; central conical stylet surrounded by 12 hooks arranged in circle; each hook with pointed base and curved end (Fig. 1E).

Ovary pre-testicular, longitudinally elongated, inverted U-shaped (Fig. 2). Oviduct arising from distal end of ovary and connecting common vitelline duct reservoir and oötype. Mehlis’ gland at base of oötype. Uterus originating from oötype and extending to genital atrium. Vagina not observed. Vitellarium follicular, well developed, co-extensive with caeca, extending from level of genital atrium to posterior extremity of body. Vitelloducts Y-shaped, with noticeably short branches; dorsal transverse vitelloducts fused at ovary; common vitelline duct median, fairly long. Eggs fusiform with two short polar filaments (Fig. 1F).

#### 3.1.3 Hosts and distribution

*Gastrocotyle trachuri* was originally described off Brest, France (North-East Atlantic) (van Beneden & Hesse, 1863) and subsequently reported from the Atlantic, from northern and southern localities.

Currently, this gastrocotylid is widely distributed in tropical and temperate waters (see Table 2) being recorded from the North-Western Mediterranean and rarely from the South-Western Mediterranean. Records from the Central and North-Western Mediterranean are scarce and include off Italy, France, Montenegro, Spain, and Turkey. In the South-Western Mediterranean this species is known only from Tunisia and Algeria. It was frequently reported from the North Atlantic (13 records, see Table 2) and rarely from the South Atlantic (off Namibia and Angola). It occurs furthermore in North Pacific (Japan, South China Sea, East China Sea, Yellow Sea and Hawaii) and the South Pacific (Australia and Tasman Sea). In the Indian Ocean, the only records of *G. trachuri* are those of Lebedev and Parukhin (see Table 2). It is likely that the carangid hosts, being vastly migratory and widely distributed in Mediterranean and oceanic waters, and the monogenean being monoxenous triggered its wide dispersal.

**Table 2:** Hosts and localities of *Gastrocotyle trachuri* van Beneden & Hesse, 1863

Species	Locality	Reference
*Trachurus trachurus* (L.) (type-host)	NE Atlantic, off France	van Beneden & Hesse (1863)
	NE and EC Atlantic, off the coast of Morocco to South-West Norway	MacKenzie et al. (2004, 2008)
	Throughout the Mediterranean	MacKenzie et al. (2004, 2008)
	NE Atlantic	Rego (1987); López-Román & De Armas Hernández (1989); Palm et al., (1999)
	NE Atlantic, Meteor Bank, off Western Sahara	Costa et al. (2012)
	SE Atlantic, off Namibia	Piasecki (1982)
	NE Atlantic, off Plymouth	Baylis & Jones (1933); Jones (1933); Sproston (1946); Llewellyn (1956, 1959, 1962, 1983); Shaw (1979); Rahemo (2012)
	NE Atlantic, off Portugal	Ângelo (2011)
	NE Atlantic, North Sea	Nicoll (1914); Naidenova & Mordvinova (1997); Campbell (2008)
	SE Atlantic	Gaevskaya & Kovaleva (1979)
	NE Atlantic, off Celtic Sea	Gaevskaya and Kovaleva, 1979, Gaevskaya and Kovaleva, 1980
	NE Atlantic, off Bay of Biscay	Gaevskaya & Kovaleva (1980, 1985)
	NE Atlantic, off South-West Ireland	Gaevskaya & Kovaleva (1985)
	EC Atlantic, Strait of Gibraltar and off Western Sahara	Gaevskaya & Kovaleva (1985)
	SE Atlantic, off Angola	Gaevskaya & Kovaleva (1985)
	Mediterranean	Euzet et al. (1993); Campbell (2008)
	Central Mediterranean, off Italy	Parona & Perugia (1889); Palombi (1949); Orecchia & Paggi (1978); Strona et al. (2010)
	NW Mediterranean, off France	Lambert (1978); Jovelin & Justine (2001)
	SW Mediterranean, off Tunisia	Feki et al. (2016)
	SW Mediterranean, off Algeria	Ichalal et al. (2017); present study
	NW Mediterranean	MacKenzie et al., 2004, MacKenzie et al., 2008
	NW Pacific, off Japan	Yamaguti (1938, 1942)
	NW Pacific, South China Sea	Parukhin (1976)
	Indian Ocean	Parukhin (1976); Reimer (1990)
*T. mediterraneus* (Steindachner)	Mediterranean	Euzet et al. (1993)
	Central Mediterranean, off Montenegro	Radujkovic & Euzet (1989)
	NW Mediterranean, off France	Mollaret et al. (2000)
	NE Mediterranean, off Turkey	Akmirza (2013)
	NW Mediterranean, off Spain	Fernandez-Jover et al. (2010)
*T. picturatus* (Bowdich)	NE Atlantic	MacKenzie et al., 2004, MacKenzie et al., 2008; Costa et al. (2012)
	SE Atlantic	Gaevskaya & Kovaleva (1979)
	NE Atlantic, off Portugal, North Sea	Gaevskaya & Kovaleva (1985)
	EC Atlantic, off Western Sahara	Gaevskaya & Kovaleva (1985)
	EC Atlantic, off Madeira	Hamdi al. (2019)
	SW Mediterranean, off Tunisia	Hamdi al. (2019)
*T. lathami* Nichols	WC Atlantic, off Venezuela	Nasir & Fuentes Zambrano (1983)
	SW Atlantic	Braicovich et al. (2012)
*T. novaezelandiae* Richardson	SE Pacific, off Australia, Tasman Sea	Lebedev (1968)
*T. indicus* (Necrasov)	Indian Ocean	Parukhin (1976)
*T. capensis* Castelnau	SE Atlantic	Gaevskaya & Kovaleva (1979)
	SE Atlantic, off Namibia	Gaevskaya & Kovaleva (1985)
	SE Atlantic, off Angola	Le Roux (2013)
*Trachurus trecae* Cadenat	SE Atlantic	Gaevskaya & Kovaleva (1979)
*Trachurus* spp.	NE Pacific (East China Sea, Yellow Sea, South China Sea)	Zhang et al. (2003)
*Selar crumenophtalmus* Bloch	Eastern Indian Ocean, off India	Parukhin (1976)
*Selar crumenophtalmus*	EC Pacific, off Hawaii	Yamaguti (1968)
*Decapterus* sp.	Eastern Indian Ocean, off India	Parukhin (1976)
*Decapterus russelli* (Rüppell)^a^	SW Indian Ocean	Parukhin (1988)
*D. maruadsi* (Temminck & Schlegel)	SW Indian Ocean	Parukhin (1988)
*Selar boops* (Cuvier)	SW Indian Ocean	Parukhin (1988)

*Abbreviations*: NE, North-Eastern; NW, North-Western; SE, South-Eastern; SW, South-Western; WC, Western-Central; EC, Eastern-Central.

a Reported as *Decapterus lajang* Bleeker.

*Gastrocotyle trachuri* is known only from carangids. First described based on material from *T. trachurus* (see van Beneden & Hesse, 1863), this species has frequently been reported from seven other congeneric hosts, *T. mediterraneus*, *T. picturatus*, *T. lathami*, *T. novaezelandiae*, *T. indicus*, *T. capensis* and *T. trecae*, *T. trachurus* and other *Trachurus* spp. It occurs allegedly on other carangid scads, *Selar crumenophtalmus* and *S. boops* (see Parukhin, 1976, 1988), and *Decapterus russelli* and *D. maruadsi* (see Parukhin, 1988).

### *3.2* *Cemocotyle* cf*. trachuri* Dillon & Hargis, 1965

#### 3.2.1 Taxonomic summary

*Host*: *Trachurus trachurus* (L.) (present study).

*Locality*: Off Bouharoun, Algeria, South-Western Mediterranean (present study).

*Site on host*: Gills.

*Voucher material*: 14 voucher specimens of *C. cf. trachuri* are deposited in the collections of the Muséum National d’Histoire Naturelle, Paris, France (MNHN HEL1545–HEL1560).

#### 3.2.2 Description

**Fig. 4:**
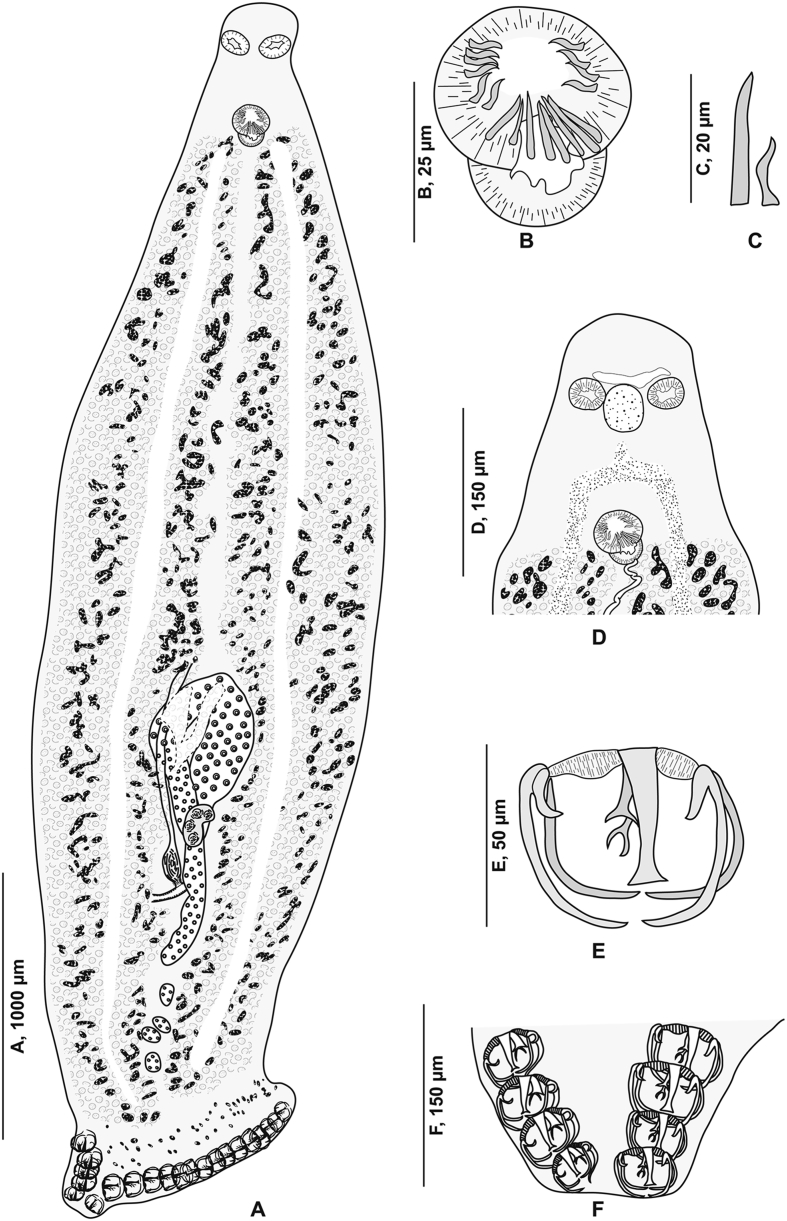
*Cemocotyle* cf. *trachuri* ex *Trachurus mediterraneus*. **A** Body, total view (MNHN HEL1547). **B** Male copulatory organ (MNHN HEL1546). **C** Atrial hook (MNHN HEL1546). **D** Anterior extremity showing the relative position of prohaptoral suckers, pharynx and male copulatory organ (MNHN HEL1546). **E** Clamp, ventral view (MNHN HEL1545). **F**, Haptor (MNHN HEL1546).

**Fig. 5:**
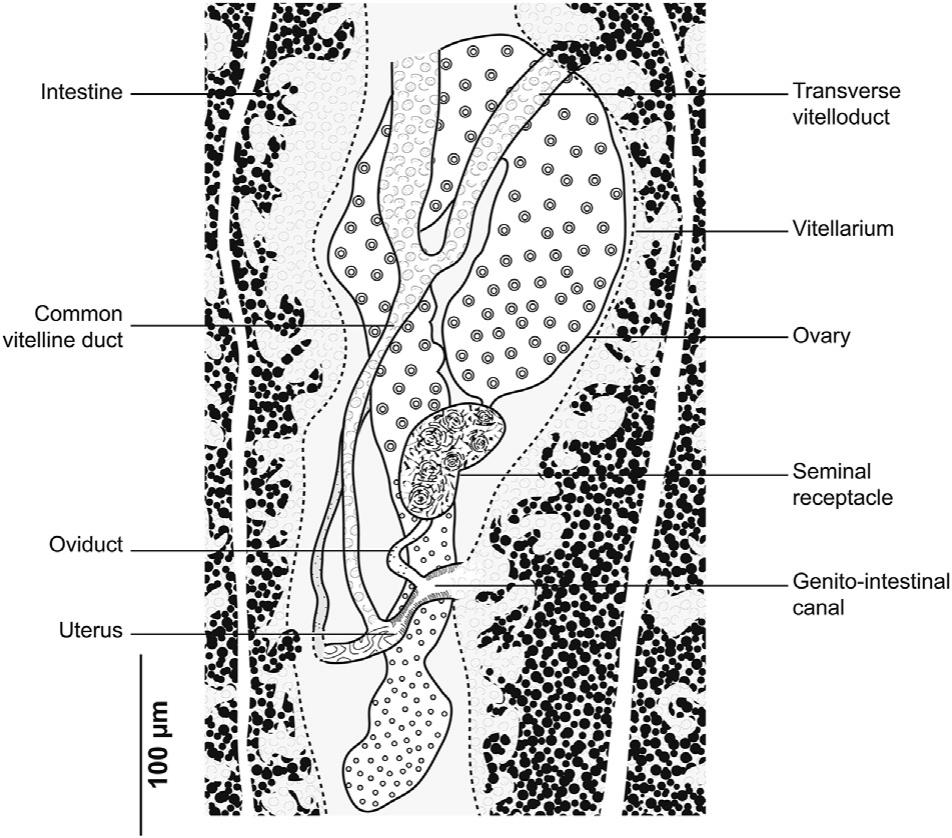
*Cemocotyle* cf. *trachuri* ex *Trachurus mediterraneus*. Detail of the reproductive organs in the region of ovary (MNHN HEL1547).

**Fig. 6:**
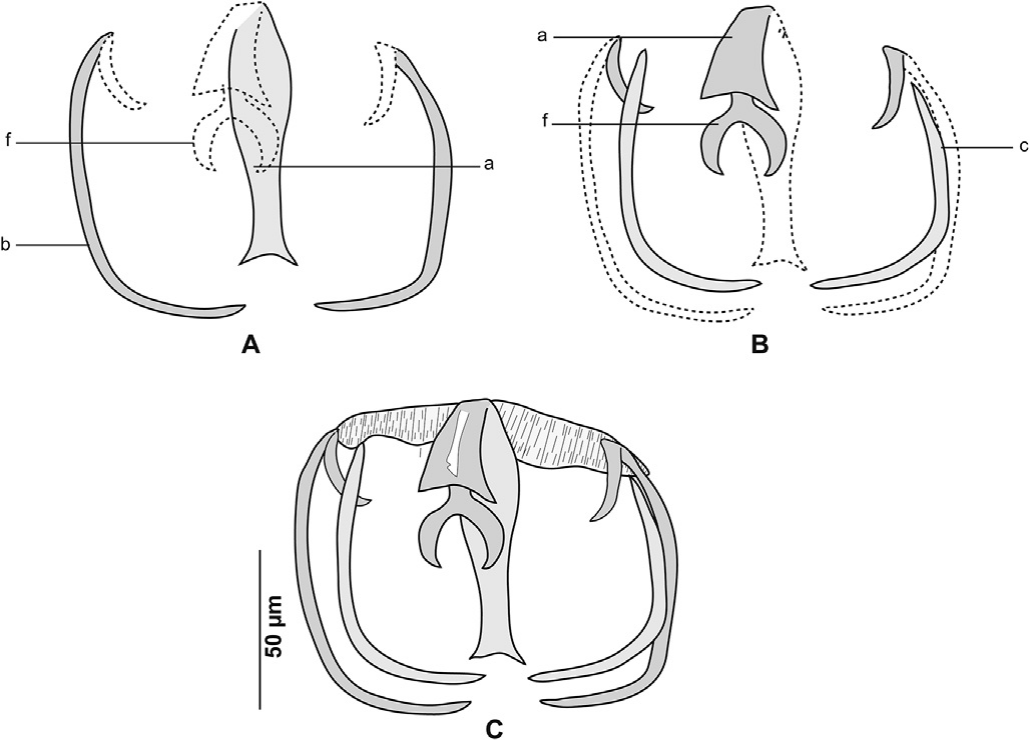
*Cemocotyle* cf. *trachuri* from *Trachurus mediterraneus*. **A** Ventral jaw. **B** Dorsal jaw. **C** Clamp, dorsal view (MNHN HEL1545).

[Based on 16 specimens; Fig. 4, Fig. 5, Fig. 6; Table 3] Body stocky, haptor considerably smaller (Fig. 4A), asymmetrical, triangular, armed with numerous clamps distributed in 2 unequal lateral rows (Fig. 4F); clamps of “muzzle” type (Fig. 4E). Ventral arm of median spring *a* long, enlarged in its proximal part and Y-shaped, with very short, barely visible branches. Sclerites of ventral jaw *b* asymmetrical; right sclerite longer than left sclerite, resulting in slight clamp asymmetry (Fig. 6A). Dorsal arm of median spring short, ending by slightly prominent T. Sclerotised piece *f* articulated at dorsal distal base of *a*. Sclerites of dorsal jaw *c* with the same asymmetry as anterior jaw (Fig. 6B); asymmetry induced by right sclerite longer than left sclerite *c* (Fig. 6C).

**Table 3:** Measurements of *Cemocotyle trachuri* from various localities

Host	*T. trachurus*	*T. novaezelandiae*	*T. trachurus*
Locality	SW Mediterranean, off Algeria	SW Pacific, off New Zealand	SE Atlantic, off Namibia
Source	Present study	Dillon & Hargis (1965)	Piasecki (1982)
Body length	1,540–3,490 (2,455; *n* = 14)	2,680–3,580 (3,280)	
Haptor length	340–650 (495; *n* = 15)	700–790 (740)	
Body width	320–850 (525; *n* = 16)	400–650 (520)	
Clamp number	25–34 (32; *n* = 13)	28–32 (30)	30
Clamp length	43–68 (51; *n* = 13)		32–77
Clamp width	20–46 (35; *n* = 13)		33–78
Hamulus length		29–40 (35)	45
Posterior hook length		22–28 (25)	
Prohaptoral sucker length	21–35 (28; *n* = 13)	34–41 (37)	
Prohaptoral sucker width	24–39 (31; *n* = 13)	37–39 (38)	
Pharynx length	20–45 (36; *n* = 13)	41–48 (44)	
Pharynx width	18–45 (34; *n* = 13)	39–47 (42)	
Distance genital atrium to anterior extremity	150–260 (220; *n* = 13)		
Genital atrium length	26–55 (37; *n* = 15)	39–57 (48)	33^a^
Genital atrium width	31–52 (41; *n* = 15)	44–62 (51)	
Long atrial spines length	14–15 (15; *n* = 12)		
Short atrial spines length	8–11 (9; *n* = 12)		
Number of testes		11–17	
Egg length		177–217 (199)	260
Egg width		72–116 (89)	

*Abbreviations*: SE, South-Eastern; SW, South-Western.

a Diameter.

Prohaptoral suckers oval, smooth-edged, opening ventrally in anterior part of body, widely separated from each other. Pharynx muscular, subspherical. Oesophagus short. Intestinal bifurcation immediately in front of genital atrium. Caeca extend to posterior extremity of body, not confluent, do not extend into haptor.

Testes *c.*11 in number, follicular, post-ovarian, intercaecal; few testes obscured by vitellarium and difficult to observe and count. Vas deferens runs upwards to join ejaculatory bulb (Fig. 4D). Genital atrium armed with numerous hooks (Fig. 4C) of different sizes and shapes arranged in anterolateral part of atrium (Fig. 4B).

Ovary elongated, pretesticular, originates in mid-posterior third of body (Fig. 5), extends forward, forms a loop, and then descends to lead into oviduct. Oviduct passing dorsally to seminal receptacle and joining genito-intestinal canal. Genito-intestinal canal abutting into right intestinal branch. Uterus dorsal to ovary, extending anteriorly to genital atrium. Seminal receptacle visible, ventrally to oviduct. Vitellarium follicular, well developed, extending in 2 lateral fields from posterior level of genital atrium to haptor. Posterior extremities of vitelline fields asymmetrical; left field slightly longer. Vitelline reservoir Y-shaped; ventral transverse vitelloducts thick, fused on midline; common vitelline duct short and thick.

#### 3.2.3 Hosts and distribution

First described off South Island, New Zealand (South-West Pacific) (Dillon & Hargis, 1965), *C. trachuri* was subsequently reported only from South-East Pacific off Chile and Peru (Oliva, 1999). This species has also been reported from Atlantic waters: a few records from the North-East Atlantic and a single record from the South-East Atlantic (off Namibia). In the Mediterranean, *C. trachuri* was reported from off France, Tunisia and Algeria (see Table 4). Note that there are no records of this species in the Indian Ocean, suggesting the presence of separated stocks of fish hosts.

**Table 4:** Hosts (*Trachurus* spp.) and localities of *Cemocotyle trachuri* Dillon & Hargis, 1965

Species	Locality	Reference
*T. novaezelandiae* Richardson (type-host)	SW Pacific, off New Zealand	Dillon & Hargis (1965)
*T. trachurus* (L.)	NE and EC Atlantic, off the coast of Morocco to south-west Norway	MacKenzie et al. (2004, 2008)
	Throughout the Mediterranean	MacKenzie et al. (2004, 2008)
	SW Mediterranean, off Tunisia	Feki et al. (2016)
	SW Mediterranean, off Algeria	Ichalal et al. (2017); present study
	SE Atlantic	Gaevskaya & Kovaleva (1979)
	NE Atlantic, North Sea	Gaevskaya & Kovaleva (1980)
	EC Atlantic, Strait of Gibraltar	Gaevskaya & Kovaleva (1985)
*T. mediterraneus* (Steindachner)	NW Mediterranean, off France	Euzet et al. (1993)
*T. picturatus* (Bowdich)	NE Atlantic, off Portugal	Costa et al. (2012)
	EC Atlantic, off Madeira	Hamdi et al. (2019)
	SE Atlantic	Gaevskaa & Kovaleva (1979)
	NE Atlantic (off Portugal, North Sea)	Gaevskaya & Kovaleva (1985)
	EC Atlantic, off Western Sahara	Gaevskaya & Kovaleva (1985)
	SW Mediterranean, off Tunisia	Hamdi et al. (2019)
*T. murphyi* Mann	SE Pacific, off Chile and Peru	Oliva (1999)
*T. capensis* Castelnau	SE Atlantic, off Namibia	Le Roux (2013); Piasecki (1982)
	SE Atlantic	Gaevskaya & Kovaleva (1979)
	SE Atlantic, off Namibia	Gaevskaya & Kovaleva (1985)
*T. trecae* Cadenat	SE Atlantic	Gaevskaya & Kovaleva (1979)
*Trachurus* spp.	EC and SE Atlantic, the length of the African continent to Namibia	Gaevskaya & Kovaleva (1985)

*Abbreviations*: NE, North-Eastern; NW, North-Western; SE, South-Eastern; SW, South-Western; EC, Eastern-Central.

*Cemocotyle trachuri* is most likely a stenoxenous polyopisthocotylean, occurring only on carangids of the genus *Trachurus* Rafinesque (Table 4). Up to now, it was reported on seven different species: *Trachurus novaezelandiae* (type-host), *T. trachurus*, *T. mediterraneus*, *T. picturatus*, *T. murphyi*, *T. capensis* and *T. trecae* (see Table 4). In the present study, *Cemocotyle* cf. *trachuri* was rare and frequently observed in association with *G. trachuri*, which suggests a recent host switch or a potential competitive between the two species.

### *3.3* *Zeuxapta seriolae* (Meserve, 1938)

#### 3.3.1 Taxonomic summary

*Type-host*: *Seriola quinqueradiata* Temminck & Schlegel [1, 6, 27].

*Other hosts*: *Seriola lalandi* Valenciennes (syn. *Seriola dorsalis* (Gill); see WoRMS, 2021) [2, 4, 8, 14, 16, 18–22, 24, 26, 27, 30, 33–38]; *S. dumerili* (Risso) [7, 9–11, 13, 15, 23, 29, 31, 32, 39]; *Seriola hippos* Günther [5]; *Seriola* spp. [12, 17, 25]; *Caranx hippos* (L.) [3, 28].

*Type-locality*: NW Pacific, off Japan [1].

*Additional localities*: Pacific [2–9, 11, 12, 14, 16–22, 24–27, 29, 30, 33–36, 38]; Atlantic [28, 37]; Mediterranean [10, 13, 15, 23, 31, 32, 39; present study].

*References*: [1] Ishii & Sawada (1938); [2] Meserve (1938); [3] Lamothe-Argumedo (1970); [4] Rohde (1978); [5] Rohde (1981 in Whittington & Chisholm, 2008); [6] Egusa (1983); [7] Ogawa & Fukudome (1994); [8] Rohde (1997); [9] Ogawa & Yokoyama (1998); [10] De Liberato et al. (2000); [11] Anshary & Ogawa (2001); [12] Ernst et al. (2002); [13] Grau et al. (2003); [14] Sharp et al. (2003); [15] Montero et al. (2004); [16] Sharp et al. (2004); [17] Chambers & Ernst (2005); [18] Mansell et al. (2005); [19] Tubbs et al. (2005); [20] Mooney et al. (2006); [21] Tubbs & Tingle (2006a, b); [22] Hutson et al. (2007a, b, c); [23] Lia et al. (2007); [24] Williams et al. (2007); [25] Whittington & Chisholm (2008); [26] Leef & Lee (2009); [27] Williams (2010); [28] Boada et al. (2012); [29] Lu et al. (2012); [30] Stuart & Drawbridge (2013); [31] Repullés-Albelda et al. (2013); [32] Öktener (2014); [33] Caraguel al. (2016); [34] Sepúlveda et al. (2016); [35] Fensham et al. (2018); [36] Vivanco-Aranda et al. (2019); [37] Camargo & Santos (2020); [38] Ingelbrecht et al. (2020); [39] Rigos et al. (2021).

*Descriptions*: [1–4; present study].

*Site on host*: Gills.

*Voucher material*: 8 voucher specimens are deposited in the collections of the Muséum National d’Histoire Naturelle, Paris, France (MNHN HEL1561–HEL1571).

#### 3.3.2 Description

**Fig. 7:**
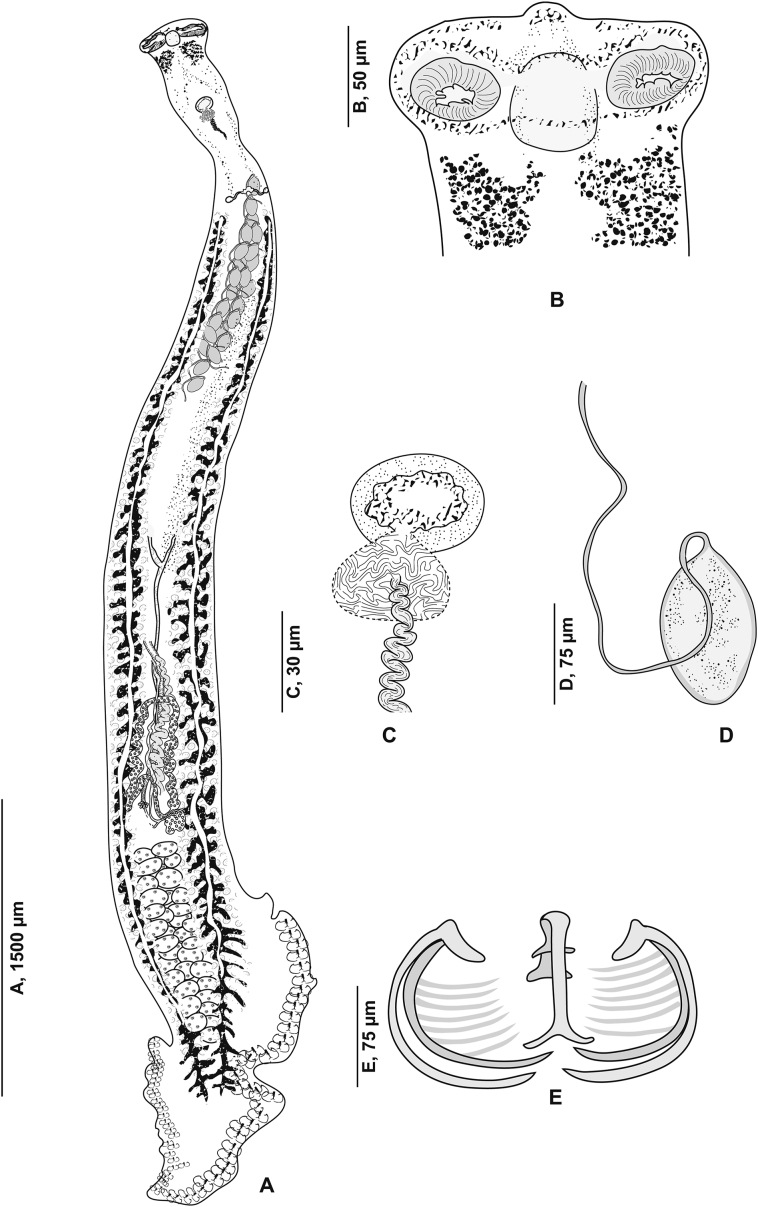
*Zeuxapta seriolae* ex *Seriola dumerili*. **A** Whole body (MNHN HEL1563). **B** Anterior extremity showing the relative position of prohaptoral suckers and pharynx (MNHN HEL1561). **C** Male copulatory organ (MNHN HEL1564). **D** Egg (MNHN HEL1564). **E** Clamp, ventral view (MNHN HEL1565).

**Fig. 8:**
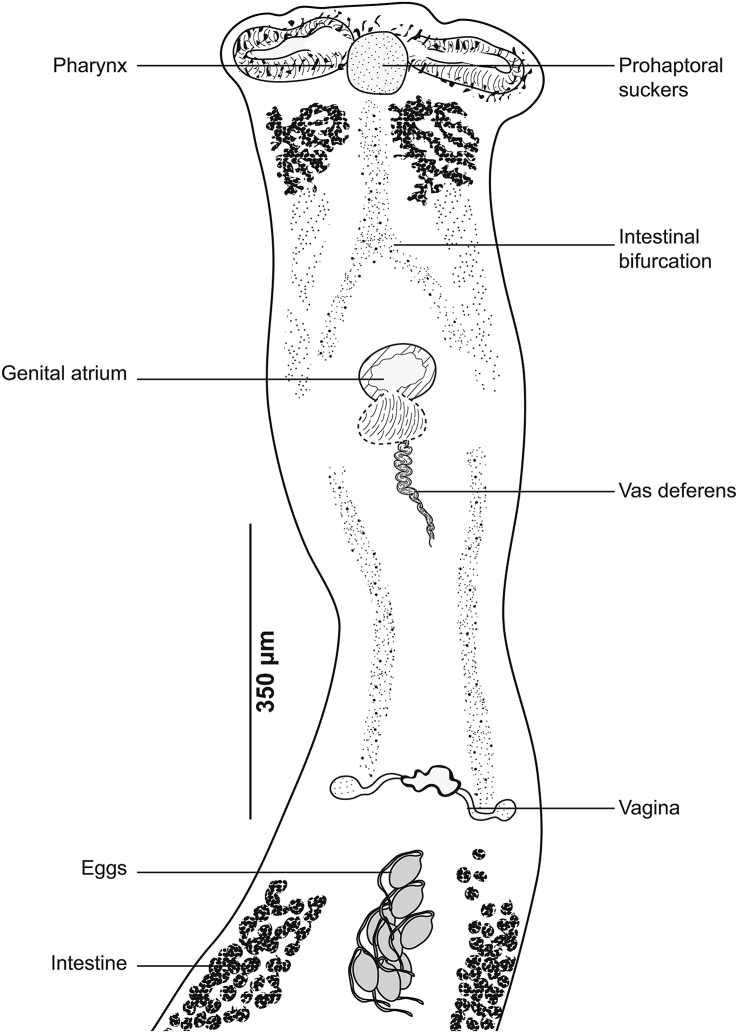
*Zeuxapta seriolae* ex *Seriola dumerili*. Anterior extremity showing the relative position of prohaptoral suckers, pharynx and male copulatory organ (MNHN HEL1562).

**Fig. 9:**
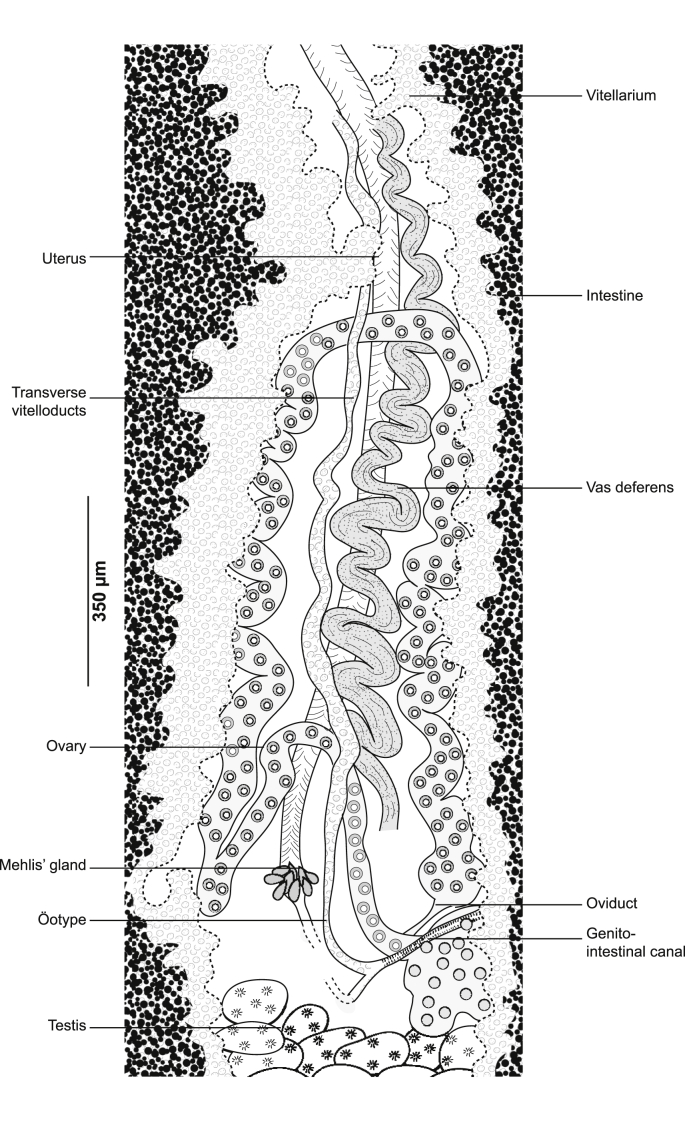
*Zeuxapta seriolae* ex *Seriola dumerili*. Detail of the reproductive organs in the region of ovary (MNHN HEL1561).

**Fig. 10:**
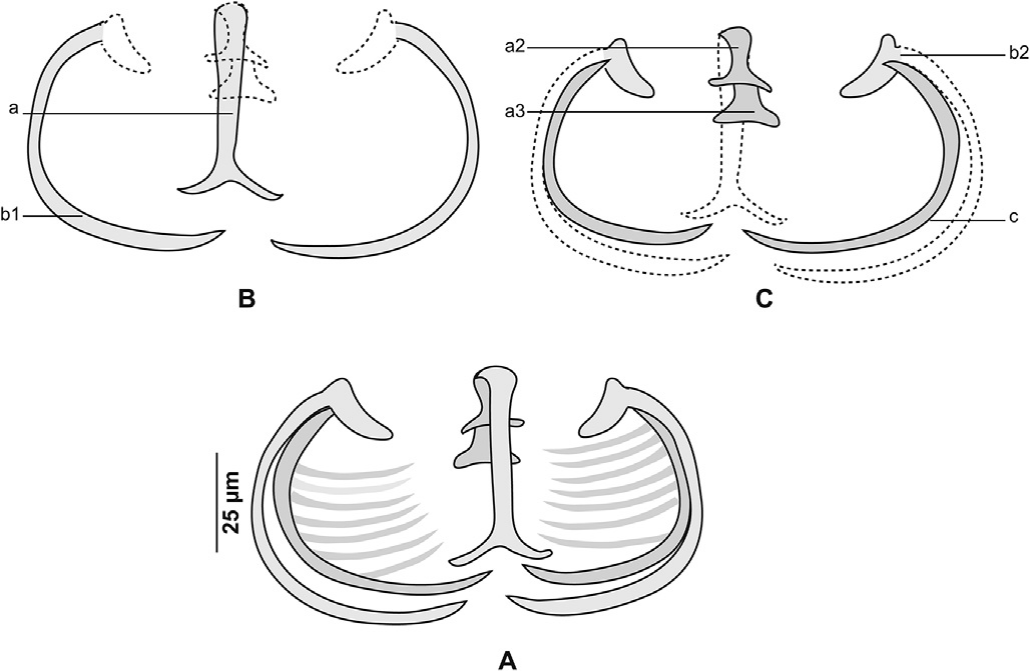
*Zeuxapta seriolae* ex *Seriola dumerili*. **A** Ventral jaw. **B** Dorsal jaw. **C** Clamp, ventral view (MNHN HEL1565).

[Based on 10 specimens; Fig. 7, Fig. 8, Fig. 9, Fig. 10; Table 5] Body flat, very elongated (Fig. 7A). Haptor triangular, leaf-like and asymmetrical, armed with 2 unequal rows of clamps. Clamps of *Microcotyle*-type (Fig. 7E). Median spring *a* long, inverted Y-shaped. Sclerites *b* of ventral jaw slightly asymmetrical (Fig. 10A); right sclerite longer than left sclerite. Dorsal arm of median spring short, T-shaped. A small sclerite F articulated ventrally on dorsal arm of median spring (Fig. 10B). Sclerites of dorsal jaw asymmetrical (Fig. 10C).

**Table 5:** Measurements of *Zeuxapta seriolae* (Meserve, 1938) from various localities

Host	*Seriola dumerili*	*Seriola quinqueradiata*	*Seriola dorsalis*		*Seriola grandis*
Locality	SW Mediterranean, off Algeria	NW Pacific, off Japan	SE Pacific, off Galapagos	Holotype	SE Pacific, off Australia
Source	Present study	Ishii & Sawada (1938)	Meserve (1938)	Rohde (1978)	Rohde (1978)
Body length (mm)	13.1–16.2 (14.1; *n* = 9)	15–20	5.1–7.5	4.9	3.2–7.5
Haptor length	3,667–9,650 (4,615; *n* = 9)				
Body width	870–1,550 (1,240; *n* = 10)	2,000	1,082–1,300	920	750–1,270
Clamp number on short side	28–42 (40; *n* = 4)	23–28	27–29	32	26–48
Clamp number on long side	30–54 (34; *n* = 4)	9–10	38–40	40	31–55
Length of clamps on short side	115–145 (132; *n* = 9)				
Width of clamps on short side	61–80 (69; *n* = 9)	498–587		108	102–130^a^
Length of clamps on long side	145–235 (202; *n* = 9)				
Width of clamps on long side	75–130 (107; *n* = 9)			165	156–195
Prohaptoral sucker length	100–180 (134; *n* = 7)	216–249	76–92	75–76	54–90
Prohaptoral sucker width	150–200 (171; *n* = 7)	149–174	120–168	112–120	54–152
Pharynx length	50–75 (62; *n* = 8)	99		39	39–47
Pharynx width	50–75 (62; *n* = 8)	66–75		40	36–43
Distance genital atrium to anterior extremity	972–1,400 (1,155; *n* = 9)			610	430–750
Genital atrium length	144–218 (172; *n* = 7)				
Genital atrium width	114–207 (161; *n* = 8)				
Distance vagina to anterior extremity	1,711–2,495 (2,095; *n* = 7)				
Number of testes	90–130 (104; *n* = 4)		93–105	120–140	95–125
Egg length	102–145 (120; *n* = 10)	149–166	96–136		
Egg width	55–88 (69; *n* = 10)	83–99	56–68		

*Abbreviations*: SE, South-Eastern; SW, South-Western; NW, North-Western.

a Diameter.

Prohaptoral suckers 2, elongated, oval and aseptate (Fig. 7B). Pharynx subspherical. Oesophagus long, diverticulated, surrounded by lateral glandular masses. Intestinal bifurcation anterior to genital atrium.

Testes numerous, *c.*80 in number, located in intercaecal field. Vas deferens conspicuous, running anteriorly to male copulatory organ where it swells into ejaculatory bulb (Fig. 7C). Genital atrium ventral, muscular, with muscle fibres. Genital atrium unarmed (Fig. 7C), subdivided into 2 cubicles by fine longitudinal septum. Cirrus unarmed.

Ovary large, occupying middle third of body (Fig. 9). Oviduct detaching from ovary, receiving vitelline reservoir and genito-intestinal canal. Genito-intestinal canal latter abutting into left intestinal branch. Oötype barely visible. Mehlis’ gland not observed. Uterus extending dorsally along body midline.

Vitellarium surrounding caeca, extending into 2 lateral fields from level of vagina to haptor. Posterior extremities of vitelline fields symmetrical, partially extending into haptor peduncle. Transverse vitelloducts not observed. Common vitelline duct exceptionally long. Vagina middorsal, at level of anterior constriction of body (Fig. 8), divided into 2 openings. Eggs subspherical, each with a long polar filament (Fig. 7D).

#### 3.3.3 Hosts and distribution

*Zeuxapta seriolae* has a wide geographical distribution in both farmed and wild fish, and was reported from the Pacific and Atlantic oceans, and the Mediterranean. First described off Japan (NW Pacific), *Z. seriolae* was reported off Taiwan, Japan, China, California, Mexico, San Diego, Australia, Galapagos Island, Chile and New Zealand. *Zeuxapta seriolae* occurs also in the Atlantic (reported only off Brazil and Venezuela). From Mediterranean waters, the species was reported from off Greece, Italy, Spain and Turkey (see Table 6). This the first report of *Z. seriolae* off Algeria. We note that this polyopisthocotylean had never been reported in the Indian Ocean. The wide spatial distribution of this monogenean is likely linked to the host mobility (the hosts species being highly migratory and sympatric).

**Table 6:** Hosts and localities of *Zeuxapta seriolae* (Meserve, 1938)

Species	Locality	Reference
*Seriola quinqueradiata* Temminck & Schlegel (type-host)	NW Pacific, off Japan; SW Pacific, off Australia	Ishii & Sawada (1938); Egusa (1983); Williams (2010)
*Seriola lalandi* Valenciennes^a^	SE Pacific, off Galapagos	Meserve (1938)
	SE Pacific, off Chile	Sepúlveda et al. (2016)
	SW Pacific, off New Zealand	Sharp et al. (2003, 2004); Mansell et al. (2005); Mooney et al. (2006); Tubbs et al. (2005); Tubbs & Tingle (2006a, b)
	SW Pacific, off Australia	Hutson et al. (2007a, b, c); Williams et al. (2007); Leef & Lee (2009); Williams (2010); Caraguel al. (2016); Fensham et al. (2018); Ingelbrecht et al. (2020)
	NE Pacific, off California	Stuart & Drawbridge (2013)
	EC Pacific, off Mexico	Vivanco-Aranda et al. (2019)
	NW Atlantic, off Brazil	Camargo & Santos (2020)
	NE Pacific, off San Diego	Stuart & Drawbridge (2013)
*Seriola lalandi*^b^	SW Pacific, off Australia	Rohde (1978, 1997)
*Seriola dumerili* Castelnau	NW Mediterranean, off Spain	Grau et al. (2003); Montero et al. (2004); Repullés-Albelda et al. (2013)
	NE Mediterranean, off Greece	Rigos et al. (2021)
	NW Mediterranean, off Italy	De Liberato et al. (2000); Lia et al. (2007)
	Eastern Mediterranean, off Turkey	Öktener (2014)
	SW Mediterranean, off Algeria	Present study
	NW Pacific, off China	Lu et al. (2012)
	NW Pacific, off Japan	Anshary & Ogawa (2001) ; Ogawa & Fukudome (1994); Ogawa & Yokoyama (1998)
*Seriola hippos* Günther	SW Pacific, off Australia	Whittington & Chisholm (2008)
*Seriola* spp.	SW Pacific, off Australia	Ernst et al. (2002); Chambers & Ernst (2005); Whittington & Chisholm (2008)
*Caranx hippos* (L.)	NW Atlantic, off Venezuela	Boada et al. (2012)
	EC Pacific, off Mexico	Lamothe-Argumedo (1970)

*Abbreviations*: NE, North-Eastern; NW, North-Western; SE, South-Eastern; SW, South-Western; EC, Eastern-Central.

a Reported as *S. dorsalis* (Gill).

b Reported as *S. grandis* Castelnau.

The host range of *Z. seriolae* is limited to two carangid genera: *Seriola* Cuvier and *Caranx* Lacépède. First described from *Seriola quinqueradiata* (Ishii & Sawada), this species was reported from *Seriola lalandi* Valenciennes (syn. *S. dorsalis* (Gill)), *S. grandis* Castelnau and *S. dumerili* Castelnau (see Table 6) and was also recorded on *Caranx hippos* (L.) (Boada et al., 2012). In the present study, this species was solely observed on its host *S. dumerili* and no other monogeneans were collected from this host.

### *3.4* *Pyragraphorus hollisae* Euzet & Ktari, 1970

#### 3.4.1 Taxonomic summary

*Type-host*: *Trachinotus ovatus* L. [1; present study).

*Additional hosts*: *Trachinotus rhodopus* Gill [5]; *Trachinotus blochii* (Lacépède) [6]; *Caranx caballus* Günther [7].

*Type-locality*: South-Western Mediterranean, off Tunisia [1].

*Additional localities*: Mediterranean [2, 3; present study]; Atlantic [4]; Pacific [5, 7]; Indian Ocean [6].

*References*: [1] Euzet & Ktari (1970); [2] Mollaret et al. (2000); [3] Icardo-Belmonte et al. (2017); [4] Madhi & Belghyti (2006); [5] Mendoza-Garfias et al. (2017); [6] Ramadhan et al. (2019); [7] Gallegos Navarro (2020).

*Descriptions*: [1; present study].

*Site on host*: Gills.

*Voucher material*: 18 voucher specimens are deposited in the collections of the Muséum National d’Histoire Naturelle, Paris, France (MNHN HEL1571–HEL1584).

#### 3.4.2 Description

**Fig. 11:**
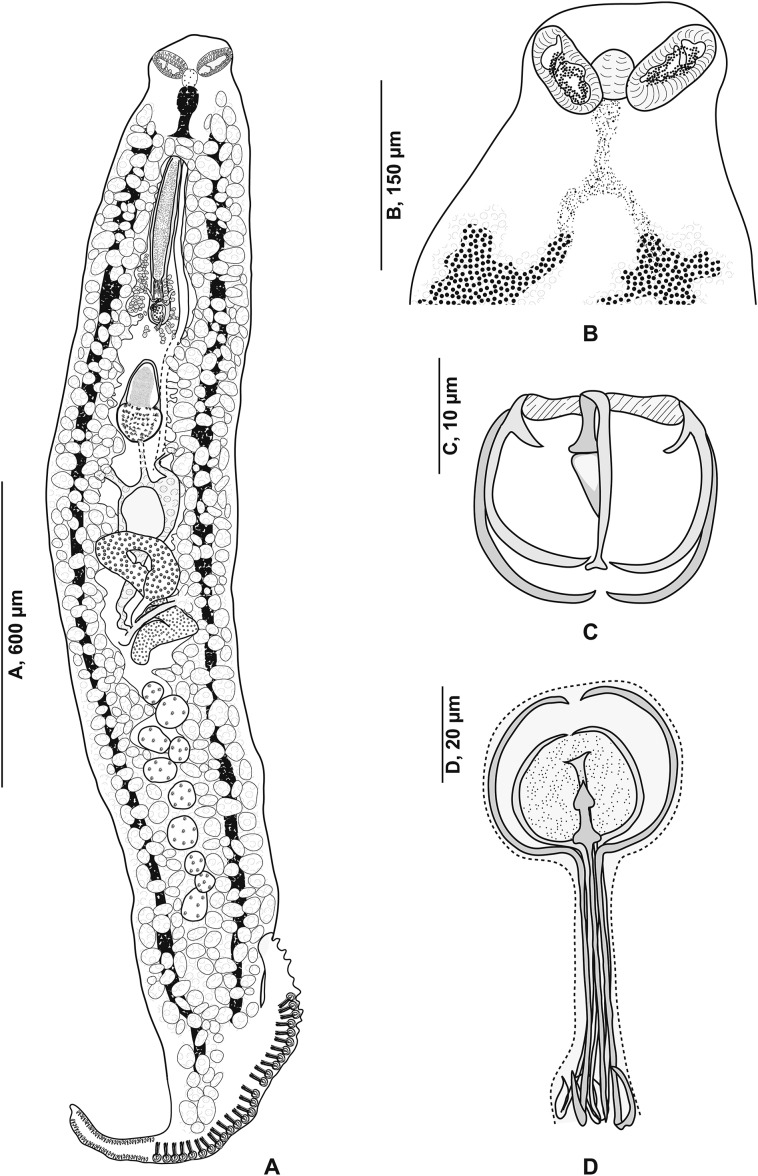
*Pyragraphorus hollisae* ex *Trachinotus ovatus*. **A** Whole body (MNHN HEL1572). **B** Anterior extremity showing the relative position of prohaptoral suckers and pharynx (MNHN HEL1573). **C***Microcotyle*-type clamp, ventral view (MNHN HEL1574). **D** “Muzzle”-type clamp, dorsal view (MNHN HEL1574).

**Fig. 12:**
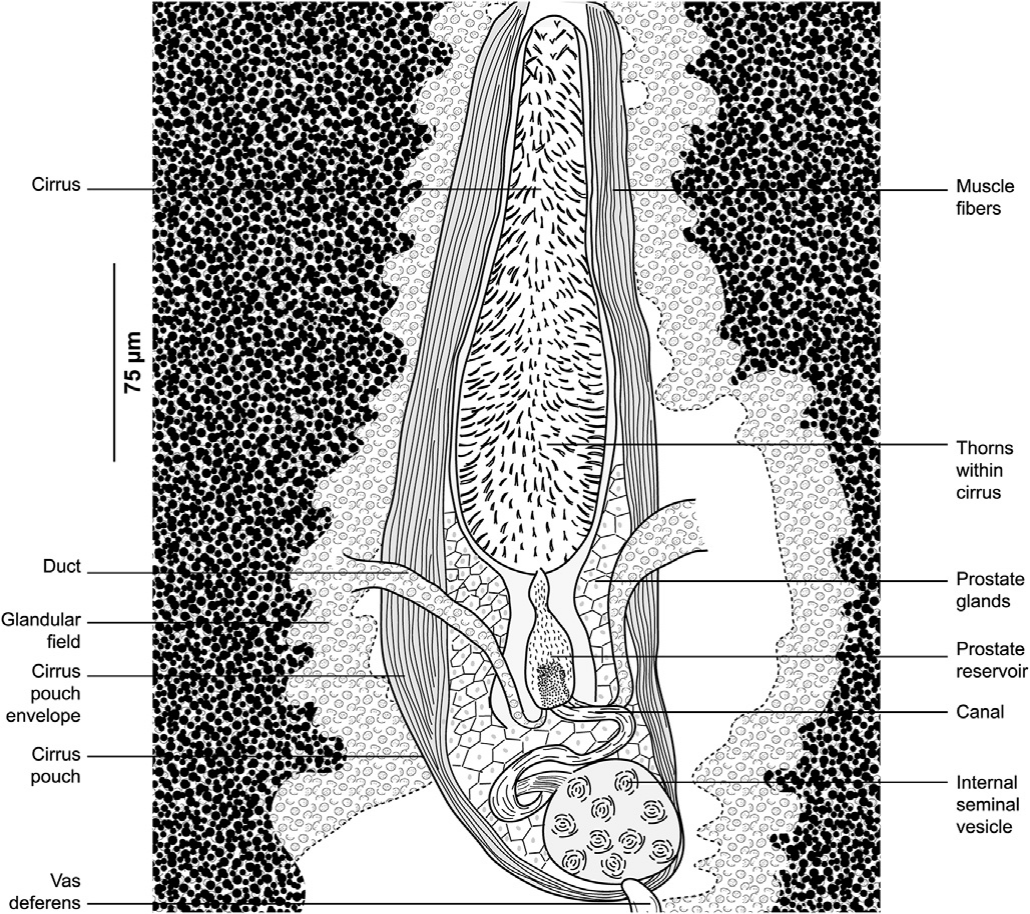
*Pyragraphorus hollisae* ex *Trachinotus ovatus*. Detail of the reproductive organs in the region of cirrus (MNHN HEL1572).

**Fig. 13:**
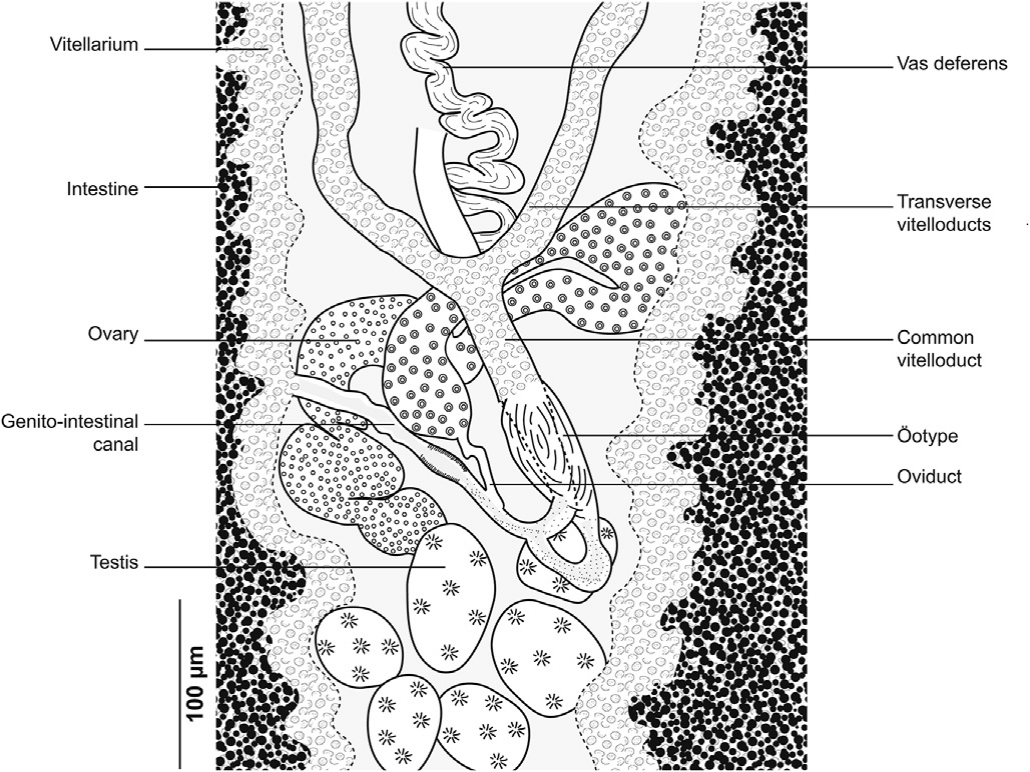
*Pyragraphorus hollisae* ex *Trachinotus ovatus*. Detail of the reproductive organs in the region of ovary (MNHN HEL1576).

**Fig. 14:**
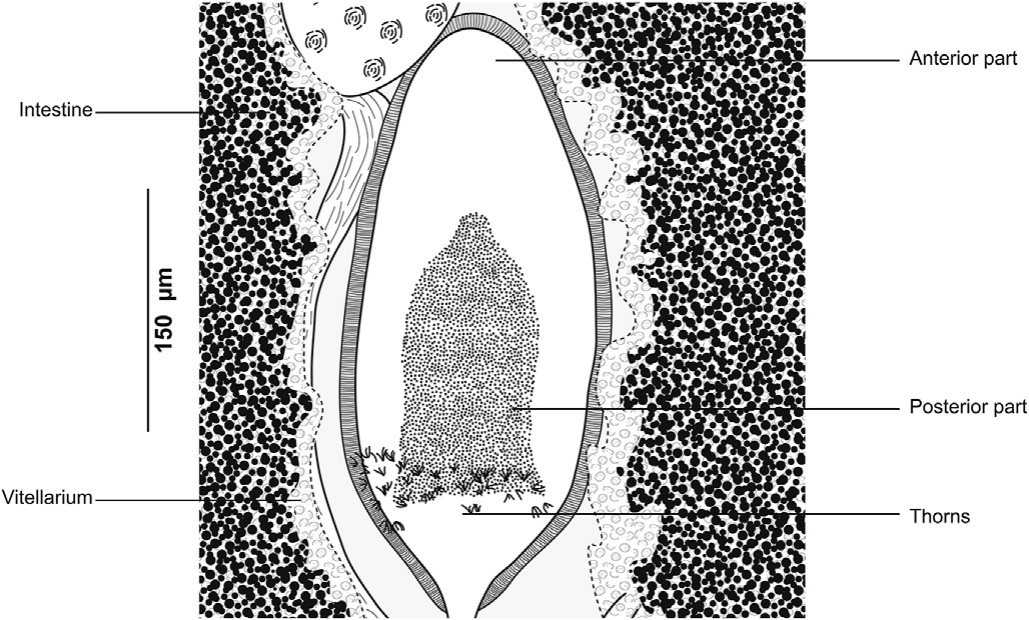
*Pyragraphorus hollisae* ex *Trachinotus ovatus*. Detail of the reproductive organs in the region of vagina (MNHN HEL1576).

**Fig. 15:**
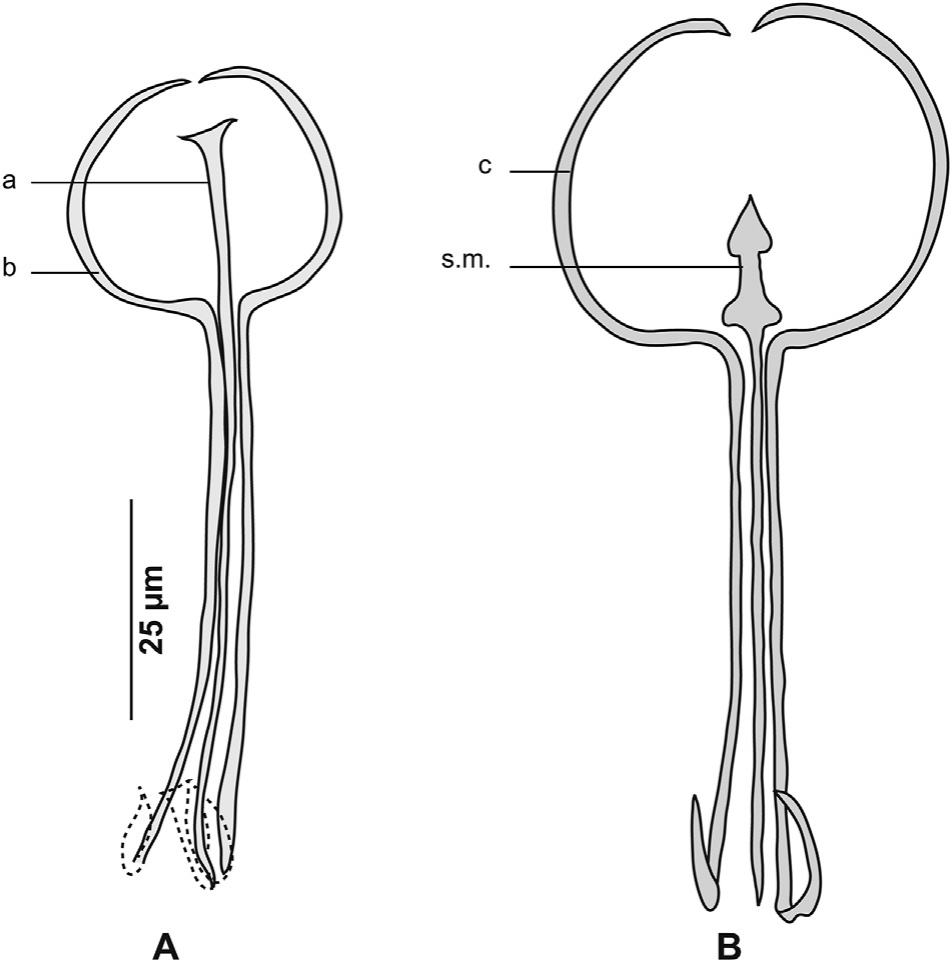
*Pyragraphorus hollisae* ex *Trachinotus ovatus*. “Muzzle”-type clamp. **A** Ventral jaw. **B** Dorsal jaw (MNHN HEL1575).

**Fig. 16:**
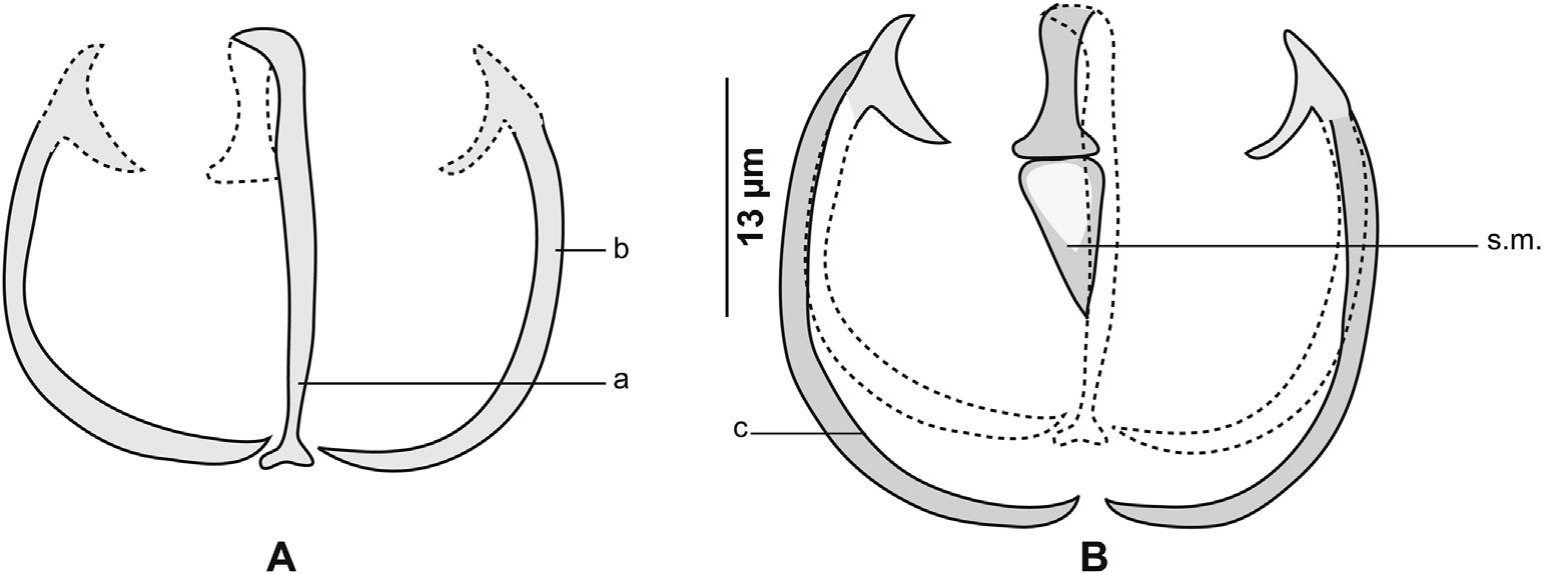
*Pyragraphorus hollisae* ex *Trachinotus ovatus*. *Microcotyle*-type clamp. **A** Ventral jaw. **B** Dorsal jaw (MNHN HEL1576).

[Based on 21 specimens; Fig. 11, Fig. 12, Fig. 13, Fig. 14, Fig. 15, Fig. 16; Table 7] Body elongated (Fig. 11A). Haptor asymmetrical, perpendicular to body axis, armed with 2 rows of clamps of *Microcotyle*-type, more or less deeply transformed, in 2 groups: stalked clamps, characteristic of the genus *Pyragraphorus* (“muzzle”-type) (Fig. 11A), elevated on stalks and occupying oblique part of the haptor; unstalked clamps small (*Microcotyle*-type) (Fig. 11A), extending to apex of haptor.

**Table 7:** Measurements of *Pyragraphorus hollisae* Euzet & Ktari, 1970 ex *Trachinotus ovatus* from South-Western Mediterranean

Locality	Off Algeria	Off Tunisia
Source	Present study	Euzet & Ktari (1970)
Body length	2,490–4,090 (3,565; *n* = 18)	2,000–2,500
Haptor length	1,265–2,735 (1,960; *n* = 17)	
Body width	366–735 (615; *n* = 21)	500–600
Clamp number (anterior series)	68–80 (76; *n* = 12)	70–84
Clamps number (posterior series)	35–45 (42; *n* = 12)	36–44
Clamp length (anterior series)	60–85 (82)	65–100
Clamp width (anterior series)	24–32 (30)	25–30
Clamp length (posterior series)	30–40 (38; *n* = 17)	30–37
Clamp width (posterior series)	20–32 (27; *n* = 17)	22–25
Prohaptoral sucker length	40–65 (54; *n* = 21)	80^a^
Prohaptoral sucker width	70–140 (114; *n* = 21)	
Pharynx length	36–65 (53; *n* = 21)	40^a^
Pharynx width	35–51 (47; *n* = 21)	
Cirrus length	218–420 (323; *n* = 21)	
Distance vagina to anterior extremity	300–1,465 (1,236; *n* = 20)	800
Vagina length	220–465 (384; *n* = 20)	
Vagina width	65–232 (166; *n* = 20)	
Number of testes	10–25 (22; *n* = 12)	8–13

a Diameter.

Clamps of anterior series characteristic of the genus *Pyragraphorus* (Fig. 11D). Each clamp formed by 2 jaws of unequal size: ventral jaw (Fig. 15A) and larger posterior jaw (Fig. 15B). Ventral arm of median spring *a* long, T-shaped on proximal side. Lateral sclerites *b* of ventral jaw asymmetrical. On proximal side, sclerites *b* drawing semicircle giving front half of clamps circular appearance. Sclerites *b* straight and parallel to each other in posterior half of clamps. Circular anterior part of clamps marked by epidermal expansions. Dorsal arm of median spring *a* prolonged by median sclerification *f*; the latter ending in a spearhead in circular part of clamps. Lateral sclerites of dorsal jaw *c* arranged as sclerites *b.*

Clamps of posterior series relatively small, of *Microcotyle*-type (Fig. 11C). Ventral arm of median spring long, thin, ends distally in slightly prominent T (Fig. 16A). Lateral sclerites of ventral jaw *b* approaching midline distally. Dorsal arm of median spring inverted T-shaped (Fig. 16B). Median sclerite in “spearhead” *s.m*. articulated on dorsal arm of median spring. Lateral sclerites of dorsal jaw *c* arched and longer than *b*.

Pair of oval prohaptoral suckers, muscular, oval, transversely-elongated (Fig. 11B), subdivided into 2 uneven cubicles by muscle septum. Pharynx spherical, opening ventrally on midline. Oesophagus short. Intestinal bifurcation anterior to level of genital atrium. Intestinal caeca obscured by vitellarium.

Testes *c*.12 in number, subcircular to oval, few and confined to post-ovarian intercaecal field, do not reach posterior junction of vitellarium. Vas deferens sinuous, arising dorsally to right of midline, extending along body midline and abutting in cirrus-pouch. Cirrus-pouch with thick wall of muscle fibres (Fig. 12), containing internal seminal vesicle, prostatic reservoir and cirrus proper. Internal seminal vesicle subspherical, connected to channel projecting forward, reaching the base of elongated and pleated chamber, corresponding to prostate reservoir. Numerous prostatic glands distributed on either side of basal third of cirrus pocket. Two ducts originating from 2 vitellarium strips, cross cirrus-pouch wall, and open at base of prostate reservoir. Prostate reservoir thins anteriorly into narrow duct, which ends in cirrus. Cirrus lateral to uterus, long, cylindrical, armed with numerous thorns (“cils” in the French nomenclature used by Euzet & Ktari (1970)).

Ovary folded, dorsal, pretesticular (Fig. 13), originates on right side, passes to left, goes up, and describes a loop to return to right where its descending branch crosses transverse branch of immature part, before throwing itself into oviduct. Genito-intestinal canal detaches from oviduct and abuts in right intestinal branch. Oviduct first receives vitelline reservoir, then forms posterior loop, which follows a swollen oötype. Mehlisʼ gland not observed. Uterus extending along body midline, dorsally, leading into genital pore. Vagina complex (Fig. 14), with smooth anterior part and posterior part forming funnel marked at its base by cilia grouped in brushes. Posteriorly, funnel continuing through narrow, short channel ending in anterior part at point of junction of transverse vitelloducts. Vitelloducts unite again at mid-length of body, at level of ovary to form ventral vitelline reservoir opening into oviduct. Vitelline follicles globular, surrounding intestinal branches.

#### 3.4.3 Hosts and distribution

*Pyragraphorus hollisae* is a poorly known species. Described for the first time from off the southern Mediterranean coasts of Tunisia by Euzet & Ktari (1970), it was recorded in the Atlantic, off Morocco; and allegedly from the Pacific off Mexico and from the Indian Ocean (off Indonesia) (Table 8). In the Mediterranean, *P. hollisae* was also reported only off France and Spain. Algeria is a new geographical record for this monogenean.

**Table 8:** Hosts and localities of *Pyragraphorus hollisae* Euzet & Ktari, 1970

Species	Locality	Reference
*Trachinotus ovatus* L. (type-host)	SW Mediterranean, off Tunisia	Euzet & Ktari (1970)
	NW Mediterranean, off France	Mollaret et al. (2000)
	NW Mediterranean, off Spain	Icardo-Belmonte et al. (2017)
	NE Eastern Central Atlantic, off Morocco	Madhi & Belghyti (2006)
*Trachinotus rhodopus* Gill	EC Pacific, off Mexico	Mendoza-Garfias et al. (2017)
*Trachinotus blochii* (Lacépède)	Eastern Indian Ocean, off Indonesia	Ramadhan et al. (2019)
*Caranx caballus* Günther	EC Pacific, off Mexico	Gallegos Navarro (2020)

*Abbreviations*: NE, North-Eastern; NW, North-Western; SW, South-Western; EC, Eastern-Central.

*Pyragraphorus hollisae* occurs mainly on pompanos (carangids of the genus *Trachinotus*) (Table 8). In addition to the type-host *T. ovatus* (see Euzet & Ktari, 1970), *P. hollisae* was reported on *T. rhodopus* (see Mendoza-Garfias et al., 2017), *T. blochii* (see Ramadhan et al., 2019) and *C. caballus* (see Gallegos Navarro, 2020). In the present study, *P. hollisae* was observed frequently in association with *Gotocotyla acanthura* (Parona & Perugia, 1896).

## 4 Discussion

To date, 42 polyopisthocotylean species have been reported from teleosts from off Algeria (Bouguerche, 2019). Unfortunately, most of these records were included in unpublished MSc and PhD theses, making them difficult to access. In addition, the monogeneans included within are often poorly and insufficiently described and these descriptions generally lack morphometric data. In the present paper, we redescribe four polyopisthocotyleans collected from the gill filaments of three carangids from off the Algerian coast, South-Western Mediterranean: *Gastrocotyle trachuri* (Gastrocotylidae) and *Cemocotyle* cf. *trachuri* (Heteraxinidae) from the Mediterranean horse mackerel *T. mediterraneus*; *Zeuxapta seriolae* (Heteraxinidae) from the greater amberjack *S. dumerili* and *Pyragraphorus hollisae* (Pyragraphoridae) from the pompano *T. ovatus*. The following sections provide accurate comparisons the morphometric variations between Mediterranean and oceanic specimens; we also briefly discuss the taxonomic status and host specificity of the four monogeneans.

### *4.1* *Gastrocotyle trachuri* van Beneden & Hesse, 1863

Among the Gastrocotylidae, the genus *Gastrocotyle* van Beneden & Hesse, 1863 is unique due to having only one side of the haptor developed as a marginal frill bearing a row of clamps extending along one side of the body to about halfway between the anterior margin of the ovary and the vaginal pore. Currently, *Gastrocotyle* includes seven species, all parasites of fishes belonging to the Carangidae Rafinesque and Scombridae Rafinesque (Table 9). The most recently described species in this genus is *Gastrocotyle buckleyi* Gupta & Krishna, 1980 from the malabar trevally *Carangoides malabaricus* (Bloch & Schneider) (syn. *Caranx malabaricus*) off India (Gupta & Tandon, 1980). *Gastrocotyle buckleyi* exhibits marked differences compared to the congeners: an elongated triangular body separated from the haptor, oblique haptor located very far from the ovary unlike in *Gastrocotyle* spp. in which the haptor only occupies only one side of the body and extends over more than half of its length. *Gastrocotyle buckleyi* should probably be removed from *Gastrocotyle*; however, details of the male copulatory organ of this species (unavailable in the original description) are warranted to determine its taxonomic status and we propose to consider this taxon *incertae sedis* pending further studies.

**Table 9:** Hosts and localities of *Gastrocotyle* spp.

Species	Type-host	Type-locality	Reference
*Gastrocotyle trachuri* van Beneden & Hesse, 1863	*Trachurus trachurus* (L.)	NE Atlantic, off France	van Beneden & Hesse (1863)
*Gastrocotyle japonica* Ishii & Sawada, 1938^a^	*Scomber japonicus* Houttuyn	NW Pacific, off Japan	Ishii & Sawada (1938)
*Gastrocotyle indica* Subhapradha, 1951	*Alepes djedaba* (Forsskål)	Eastern Indian Ocean, off India	Subhapradha (1951)
*Gastrocotyle kurra* Unnithan, 1968	*Decapterus russelli* (Rüppell)	Eastern Indian Ocean, off India	Unnithan (1968)
*Gastrocotyle kalla* Unnithan, 1968^b^	*Alepes djedaba* (Forsskål)^c^	Eastern Indian Ocean, off India	Unnithan (1968)
*Gastrocotyle mozambiquensis* Lebedev & Galkina in Lebedev, 1975	*Decapterus* sp.	Western Indian Ocean, off Mozambique; Eastern Indian Ocean, off India	Lebedev (1986)
*Gastrocotyle buckleyi* Gupta & Krishna, 1980	*Carangoides malabaricus* (Bloch & Schneider)	Eastern Indian Ocean, off India	Gupta & Tandon (1980)

*Abbreviations*: NE, North-Eastern.

a Synonym of *Gastrocotyle trachuri* (see WoRMS, 2021).

b Synonym of *Gastrocotyle indica* (see WoRMS, 2021).

c Reported as *Caranx kalla* Cuvier.

Overall, the present specimens agree with the diagnosis of *G. trachuri*, the type-species of the genus *Gastrocotyle*, originally described on the Atlantic horse mackerel *T. trachurus* off Brest (van Beneden & Hesse, 1863). These authors provided a brief description lacking morphometric data and a poor illustration. *Gastrocotyle trachuri* was redescribed by Parona & Perugia (1889) and Jones (1933). The latter author pointed out that the number of hooks reported by van Beneden & Hesse (1863) is linked to the stage of development as they probably described immature specimens (Jones, 1933). We fully agree with this suggestion based on the examination of the present specimens (see Fig. 1H–J). Examination of 32 specimens of *G. trachuri* (Table 1) revealed that the material from off the Algerian coast agrees morphologically well with the descriptions of Jones (1933) and Radujkovic & Euzet (1989), particularly in having *a* terminal lappet armed with three pairs of hooks (Fig. 1H–J; also see figure 2C in Jones, 1933).

The description provided here adds several details, contributing to the diagnosis (clamp sclerites and male copulatory organ) and extends the known range for the morphometric data of *G. trachuri*. We provide herein for the first time, illustrated drawings of the male copulatory organ and descriptions and organisation clamp sclerites. The present specimens differ from the Atlantic specimens of *G. trachuri* ex *T. capensis* (see Piasecki, 1982) in having smaller clamps (60–75 × 38–58 *vs* 70–100 × 55–77 μm), smaller postero-lateral hooks (16–20 *vs* 25 μm), slightly reduced hamuli (35–52 *vs* 58 μm), and an apparently larger genital atrium (25–30 *vs* 16 μm).

The specimens of *G. trachuri* collected off Algeria differ from the Mediterranean specimens from *T. mediterraneus* described by Radujkovic & Euzet (1989) in having a reduced body width (610–1,025 *vs* 1,000 μm), larger postero-lateral hooks (16–20 *vs* 10 μm), and larger posterior hooks (20–26 *vs* 20 μm).

Unfortunately, only two measurements are available for comparison with the Atlantic specimens reported from off Portugal by Ângelo (2011) since the author provided only body length (3,000–4,000 μm) and clamp number (24–43).

The specimens of *G. trachuri* collected off Algeria can be distinguished from the Australian specimens (Indian Ocean) described from *T. novaezelandiae* by Lebedev (1968) in the smaller body length (2,370–3,675 *vs* 2,690–5,030 μm) and in having significantly larger clamps (60–75 × 38–58 *vs* 50–60 × 17–19 μm). The Australian specimens also have a reduced genital atrium width (10–30 *vs* 22–33 μm), while specimens from off Algeria possess fewer atrial hooks (12 *vs* 16) which are also longer (18–19 *vs* 14 μm).

The present specimens of *G. trachur*i can be differentiated from the Indian specimens (Indian ocean) described from *Decapterus* sp. by Pillai (1968) in having a greater body width (610–1,025 *vs* 500–750 μm), a greater number of clamps (30–40 *vs* 22–30) which are also larger (60–75 × 38–58 *vs* 45–50 × 60–75 μm), and a slightly smaller genital atrium (25–30 × 22–33 *vs* 30–35 × 35–35 μm).

Finally, the Mediterranean specimens of *G. trachur*i described here differ from the Atlantic specimens collected off Plymouth by Jones (1933) in having a shorter body (2,370–3,675 *vs* 4,700 μm) and a slightly shorter haptor (610–1,025 *vs* 1,200 μm), and in having larger prohaptoral suckers (22–33 × 26–39 *vs* 23 × 15 μm) and pharynx (34–54 × 30–50 *vs* 46 × 30 μm).

We note that only the specimens from the Indian ocean (ex *T. novaezelandiae* off Australia) are reported with a greater number (16) of atrial hooks (see Lebedev, 1968) whereas 12 hooks were described in all other studies (Jones, 1933; Pillai, 1968; Piasecki, 1982; Radujkovic & Euzet, 1989; Ângelo, 2011). Additionally, our careful comparison of the present morphometric data with the Mediterranean specimens described by Radujkovic & Euzet (1989) revealed a general overlap whereas both sets of Mediterranean specimens differ considerably from the oceanic specimens, from the Atlantic and Indian oceans. It is therefore possible that *G. trachuri* does not have a wide specificity nor geographical distribution but rather represents several *G. trachuri*-like species, each specific to a single host, which could not be distinguished morphologically. It is likely that future study will show that the gastrocotylid from the Mediterranean is a distinct species; this would require a detailed morphological and a molecular study of specimens from both the Mediterranean and oceanic waters.

### *4.2* *Cemocotyle* cf. *trachuri* Dillon & Hargis, 1965

The genus *Cemocotyle* Sproston, 1946 includes four species considered valid (Table 10). The type-species *Cemocotyle carangis* (MacCallum, 1913) was described based on material from the blue runner *Caranx chrisoms* (Mitchill) as *Microcotyle carangis* MacCallum, 1913 (see MacCallum, 1913). Based on the presence of modified clamps on one side of the haptor, Sproston (1946) erected the genus *Cemocotyle* to accommodate *M. carangis*. In the diagnosis of the genus, Sproston (1946) mentioned the presence of a terminal lappet and noted that the haptor did not contain vitellarium nor intestinal diverticula. However, the illustration of the general morphology by MacCallum (1913) clearly shows the presence of intestinal branches, vitellarium and testes penetrating the haptor.

**Table 10:** Hosts and localities of *Cemocotyle* spp.

Species	Reported as	Type-host	Type-locality	Reference
*C. carangis* (MacCallum, 1913) Sproston, 1946	*Microcotyle carangis* MacCallum, 1913^a^	*Caranx chrysos* (Mitchill) (Carangidae)	NW Atlantic, off Massachusetts	MacCallum (1913); Sproston (1946)
*C. borinquensis* Price, 1962		*Caranx caballus* Günther (Carangidae)^b^	WC Atlantic, off Puerto Rico	Price (1962)
*C. noveboracensis* Price, 1962	*Axine carangis* MacCallum, 1919^a^; *Axine* (*Heteraxine*) *carangis* (MacCallum, 1919) Yamaguti, 1938^a^^,^^c^	*Caranx hippos* (L.); *Caranx ruber* (Bloch) (Carangidae)	NW Atlantic, off New York	Price (1962)
*C. trachuri* Dillon & Hargis, 1965		*Trachurus novaezelandiae* Richardson (Carangidae)	SW Pacific, off New Zealand	Dillon & Hargis (1965)

*Abbreviations*: NW, North-Western; SW, South-Western; WC, Western-Central.

a Junior synonym.

b Reported as *Paratractus caballus*.

c According to Yamaguti (1938); this species is placed in the genus *Axine*, subgenus *Heteraxine* (Yamaguti, 1938).

Price (1962) indicated the presence of a terminal lappet in his description of *Cemocotyle borinquensis* Price, 1962 from the green jack *Caranx caballus* Günther (syn. *Paratractus caballus* (Risso)) off Puerto Rico, USA. After re-examination of specimens of *C. carangis* from the collection of MacCallum, Price (1962) suggested *Cemocotyle noveboracensis* (MacCallum, 1919) Price, 1962to group specimens collected on *Caranx hippos* (L.) and *Caranx ruber* (Bloch) (see Price, 1962); this author also considered that the presence of *C. carangis* in *Trachinotus carolinus* (L.) is unusual and due to an accidental parasitism. However, we remark that a *Cemocotyle* sp. has already been reported on *Trachinotus goodei* Jordan & Evermann, another carangid host of the same genus (Luque & Cézar, 2004).

In addition, the presence of a terminal lappet in *Cemocotyle* spp. has been indicated in three species: *C. carangis*, *C. borinquensis* (see Price, 1962; Kritsky et al., 2011) and *C. noveboracensis* (see Price, 1962). However, Kritsky et al. (2011) after examining the type-specimens and 52 other specimens of *C. noveboracensis* from the USA, confirmed the lack of a terminal lappet. Similarly, these authors studied several specimens of *C. carangis* from *Caranx hippos* off Puerto Rico from Museum collections and confirmed the lack of a terminal lappet and attributed these specimens to *C. noveboracensis* (see Kritsky et al., 2011). However, these authors emphasized that *C. noveboracensis* differs from all congeners in the lack of a terminal lappet and the organization of the male copulatory organ.

*Cemocotyle trachuri* was reported on various *Trachurus* spp. (see Table 4). None of the previous records included descriptions, illustrations or morphometric data. These host records are probably based on insufficient evidence and unjustifiable. Although Piasecki (1982) provided a very brief description, he included no details of internal anatomy and morphology and listed only a few measurements. Hence, an illustrated redescription along with morphometric data seemed necessary considering the dubious reports of this species and the uncertainty of its occurrence on the hosts in previous records.

Specimens of *Cemocotyle* collected on *T. trachurus* off Algeria, differ from the Pacific specimens of *C. trachuri* from *T. novaezelandiae* (see Dillon & Hargis, 1965) in having a shorter haptor (340–650 *vs* 700–790 μm), smaller prohaptoral suckers (21–35 × 24–39 *vs* 34–41 × 37–39 μm), a clearly smaller pharynx (20–45 × 18–45 *vs* 41–48 × 39–47 μm), and a smaller genital atrium (26–55 × 31–52 *vs* 39–57 × 44–62 μm). Unfortunately, clamp dimensions were not given for the Pacific specimens.

The Mediterranean specimens of *Cemocotyle* can be distinguished from *C. trachuri* (reported on the same host, *T. trachurus*) off Namibia (Piasecki, 1982) in having slightly reduced clamp width (25–34 *vs* 30 μm). However, only a few measurements for the Atlantic specimens are available for comparison (Table 3).

Details of morphology and anatomy of the specimens collected from *T. trachurus* in the present study were carefully considered and we conclude that these specimens are similar to *C. trachuri* in general morphology and internal anatomy, except with regard to the terminal lappet, which is lacking in the present material. Our specimens are clearly smaller than specimens of *C. trachuri* from *T. novaezelandiae*, but differences were not obvious; they have different hosts (*T. trachurus vs T. novaezelandiae*) and the localities are very distant (Mediterranean *vs* Pacific Ocean). However, the proposal of a new species based on the absence of terminal lappet is not desirable as it would cause additional instability to the already confused composition of the genus (Kritsky et al., 2011). Hence, pending comparison of molecular sequences from both hosts and localities, we use *Cemocotyle* cf. *trachuri* to designate the heteraxinid collected from *T. trachurus* from the South-Western Mediterranean.

We emphasize the simultaneous occurrence of four polyopisthocotylean species, i.e. *Gastrocotyle trachuri*, *Cemocotyle* cf. *trachuri*, *Pseudaxine trachuri* and *Allogastrocotyle trachuri* in the same fish host specimen. The latter two species were recently described and illustrated (Bouguerche et al., 2019b, c).

### *4.3* *Zeuxapta seriolae(*Meserve, 1938*)*

At present, the genus *Zeuxapta* includes three species considered valid (Table 11): *Z. kahala*, *Z. seriolae* and *Z. taylori*. The first two species parasitize carangids of the genus *Seriola* Cuvier and the third parasitizes the scombrid *Thunnus albacares* (Bonnaterre). Only *Z. seriolae* occurs in the Mediterranean.

**Table 11:** Hosts and localities of *Zeuxapta* spp.

Species	Reported as	Type-host	Type locality	Reference
*Z. seriolae* (Meserve, 1938) Price, 1962	*Zeuxapta japonica* Yamaguti, 1961^a^; *Zeuxapta zyxivaginata* Unnithan, 1957^a^	*Seriola lalandi* Valenciennes (Carangidae)^b^	SE Pacific, off Galapagos	Meserve (1938); Price (1962); Unnithan (1957)
*Z. kahala* (Yamaguti, 1968) Ogawa & Egusa, 1980	*Aspinatrium kahala* (Yamaguti, 1968)^a^	*Seriola dumerili* (Risso) (Carangidae)	NE Pacific, off Hawaii	Ogawa & Egusa (1980); Yamaguti (1968)
*Z. taylori* Payne, 1990		*Thunnus albacares* (Bonnaterre) (Scombridae)	NE Pacific, off California	Payne (1990)

*Abbreviations*: NE, North-Eastern; SE, South-Eastern.

a Junior synonym.

b Reported as *Seriola dorsalis*.

By the possession of an asymmetric haptor, the organization of the genital atrium and the vagina, our specimens collected on *S. dumerili* are placed within the Heteraxinidae Unnithan, 1957 and are members of the genus *Zeuxapta* Unnithan, 1957 (see Mamaev, 1990; Yamaguti, 1963). By their morpho-anatomical characters, our specimens of *Z. seriolae* are similar to those described in different regions.

However, comparison of morphometric data of the Mediterranean specimens of *Z. seriolae* from *S. dumerili* revealed that they can be differentiated from specimens collected from *S. quinqueradiata* in the Pacific (off Japan; see Ishii & Sawada, 1938) in having a slightly shorter body (13.07–16.2 *vs* 15–20 mm) and a shorter haptor (870–1,550 *vs* 2,000 μm), more clamps on short and on long side, somewhat smaller prohaptoral suckers (100–180 *vs* 216–249 μm), and a smaller pharynx (50–75 *vs* 99 μm). The Pacific specimens also have larger eggs (102–145 × 55–88 *vs* 149–166 × 83–99 μm).

The Mediterranean specimens of *Z. seriolae* differ from those collected from *S. dorsalis* off the Galapagos (Meserve, 1938) in having a significantly greater body length (13.07–16.2 mm *vs* 5,110–7,540 μm), more clamps on short and on long side, and an apparently larger prohaptoral sucker (100–180 *vs* 76–92 μm). Dimensions of clamps, pharynx, and genital atrium are not available for specimens off Galapagos (Table 5).

The Mediterranean specimens of *Z. seriolae* can be distinguished from the specimens from *S. grandis* collected in the Pacific (off Australia; see Rohde, 1978) in having an apparently greater body length (13.07–16.20 mm *vs* 3,200–7,500 μm) and body width (870–1,550 *vs* 750–1,270 μm), larger prohaptoral suckers (100–180 × 150–200 *vs* 54–90 × 54–152 μm) and larger pharynx (50–75 × 50–75 *vs* 39–47 × 36–43 μm). Specimens from Australia have fewer clamps (Table 5) but the number of clamps (and egg size) of specimens from both localities (Algeria and Australia) show overlapping ranges. Dimensions of clamps, pharynx and genital atrium were not given for the Australian specimens. We note however, that the two populations differ in the distance between the genital atrium and anterior extremity (972–1,400 *vs* 430–750 μm).

In light of the available data, we could not draw any conclusion regarding differences between the Mediterranean and Pacific specimens, as specimens from off Algeria are smaller than Pacific specimens from off Japan (Ishii & Sawada, 1938) but larger than those from off Galapagos (Meserve, 1938) and off Australia (Rohde, 1978). Probably the size of *Z. seriolae* depends on host size and some specimens from previous studies were collected from larger host individuals, as shown for another polyopisthocotylean occurring on the same host (Price, 1962; Montero et al., 2003). Similarly, we stress the importance of including data on host size in taxonomic studies of monogeneans (Montero et al., 2003). Large variation in morphometric data of *Z. seriolae* was already demonstrated by Rohde (1978). In addition, Montero et al. (2003) listed an additional species of the Heteraxininae, *Pseudoallencotyla pricey* (Kritsky, Noble & Moser, 1978), occurring on more than one host species and from a broad geographical area (Montero et al., 2003). It is likely that the long-distance migrations of the host allowed the establishment of the monogenean *Z. seriolae* in different waters. Nevertheless, it would be interesting to obtain *cox*1 sequences of these heteraxinids from the various fish host species; this would possibly lead to detection of the presence of several cryptic species.

Algeria in a new locality record for *Z. seriolae* and this finding extends the geographical range of this species to the South-Western Mediterranean. As descriptions and illustrations of clamps sclerites were not included in previous descriptions, this redescription extends the knowledge of some important taxonomic features of this monogenean.

### 4.4 *Pyragraphorus hollisae* Euzet & Ktari, 1970

Currently, *Pyragraphorus* includes only two valid species: *Pyragraphorus pyragraphorus* (MacCallum & MacCallum, 1913) and *P. hollisae* Euzet & Ktari, 1970 (WoRMS, 2021) (Table 12). All other species previously included in this genus, i.e. *Pyragraphorus incomparabilis* (MacCallum, 1916), *Pyragraphorus hippos* Hargis, 1956 and *Pyragraphorus caballeroi*Zerecero, 1960 are currently included in the genus *Allopyragraphorus* Yamaguti, 1963 (see Yamaguti, 1963). Note that all the previously mentioned species are known only from species of the Carangidae, belonging to the genera *Trachinotus* and *Caranx* (see MacCallum, 1913; 1916; Sproston, 1946; Hargis, 1956; Zerecero, 1960; Yamaguti, 1963; Euzet & Ktari, 1970).

**Table 12:** Hosts and localities of *Pyragraphorus* spp.

Species	Reported as	Type-host	Type-locality	Reference
*Pyragraphorus pyragraphorus* (MacCallum & MacCallum, 1913)	*Microcotyle pyragraphorus* MacCallum & MacCallum, 1913^a^	*Trachinotus carolinus* (L.) (Carangidae)	Probably from NW Atlantic (Sproston, 1946; Hargis, 1956)	MacCallum & MacCallum (1913); Sproston (1946)
*Pyragraphorus hollisae* Euzet & Ktari, 1970		*Trachinotus ovatus* (L.) (Carangidae)	SW Mediterranean, off Tunisia	Euzet & Ktari (1970)

*Abbreviations*: NW, North-Western; SW, South-Western.

a Junior synonym.

*Pyragraphorus* spp. are characterized by having a horizontally oriented haptor with a fish-tail appearance and armed with modified clamps; the distal half bears two rows of normal clamps and the proximal half bears two rows of modified clamps (Sproston, 1946).

The present specimens agree with the diagnosis of *P. hollisae*, originally described from the pompano *T. ovatus* collected off Tunisia, South-Western Mediterranean (Euzet & Ktari 1970) and comparison of the morphometric data for the present material with the redescription of this species provided by Euzet & Ktari (1970) did reveal a few differences (Table 7). The specimens from off Algeria differ from the specimens from off Tunisia in having a greater body length (2,490–4,090 *vs* 2,000–2,500 μm) and a slightly wider body (366–735 *vs* 500–600 μm); a wider range of variation of the distance between the vagina and anterior extremity (300–1,465 *vs* 800 μm) and a greater upper limit for the number of testes (10–25 *vs* 8–13). However, these differences are subtle and should be considered to represent intraspecific variation.

## Funding

The research leading to the results presented in this publication was partly carried out with infrastructure funded by the 10.13039/501100005307Direction Générale de la Recherche Scientifique et du Développement Technologique(DGRSDT) and by the Laboratoire de Biodiversité et Environnement: Interactions – Génomes (LBEIG), Université des Sciences et de la Technologie Houari Boumediene (USTHB), Algiers, Algeria. This study was also supported by the Institut de Systématique, Évolution, Biodiversité (ISYEB), Muséum national dʼHistoire naturelle (MNHN) Paris, France), and a framework agreement project of the DeepBlue Project: *Distance Crossborder Traineeship Programme* co-financed by the 10.13039/100014510European Maritime and Fisheries Fund (EMFF) for the analysis, interpretation of data and the writing of the manuscript. The funders had no role in study design, data collection and analysis, decision to publish, or preparation of the manuscript.

## CRediT author statement

Chahinez Bouguerche: Methodology, Writing - Original Draft, Conceptualisation, Writing, Funding acquisition. Fadila Tazerouti: Methodology, Project administration, Review. Jean-Lou Justine: Methodology, Writing - Original Draft, Conceptualisation, Writing - Review & Editing, Supervision, Project administration, Funding acquisition. All authors read and approved the final manuscript.

## Declaration of competing interests

The authors declare that they have no competing interests.
